# Factors associated with mental health stigma among teachers and caregivers of primary school children in Uganda

**DOI:** 10.1186/s12889-025-25059-z

**Published:** 2025-11-04

**Authors:** Nora Braathu, Mukisa Marjorie Kabatooro, Juliet N. Babirye, Tore Wentzel-Larsen, Ane-Marthe Solheim Skar, Ingunn M. S. Engebretsen

**Affiliations:** 1 Department of Global Public Health and Primary Care, Centre for International Health, University of Bergen, Bergen, Norway; 2 School of Public Health, College of Health Sciences, Makerere University, Kampala, Uganda; 3 Norwegian Centre for Violence and Traumatic Stress Studies (NKVTS), Oslo, Norway; 4 Norwegian Institute of Public Health, Global Health Cluster, Oslo, Norway

## Abstract

**Background:**

Uganda has one of the youngest populations in the world, with around 55% aged below 18 years. Approximately 23% of Ugandan youths are struggling with mental disorders. Despite the high prevalence of mental disorders, the existing stigma surrounding mental health may discourage individuals from seeking help, resulting in untreated conditions that may worsen over time. Examining the factors associated with stigma is crucial for understanding specific target areas to reduce its impact and prevent stigma. The current paper examines factors associated with mental health stigma among teachers and caregivers in 18 primary schools in Mbale, Uganda.

**Methods:**

The current cross-sectional study used baseline data from the TREAT INTERACT stepped-wedge cluster randomized trial among 191 teachers and 732 caregivers. Stigma was measured using the revised Perceived Devaluation-Discrimination scale (PDD). Mental health knowledge was measured with the Mental Health Knowledge Schedule (MAKS), and Psychological aggression was measured with the Dimensions of Discipline Inventory (DDI). Data were analyzed using linear mixed effects models in R. Mean scores were rescaled to range from 0 to 100.

**Results:**

Teachers had a mean of 30.7 (SD = 14.0) on the mental health stigma measure (scale 0 -100), while caregivers scored significantly higher with a mean of 36.8 (SD = 16.9). Stigma scores were analyzed as continuous variables. Lower mental health knowledge (Teachers: -0.37, 95% CI = -0.52; -0.23, Caregivers: -0.25, 95% CI = -0.33; -0.17) and higher prevalence of psychological aggression (Teachers: 0.14, 95% CI = 0.07; 0.22, Caregivers: 0.15, 95% CI = 0.10; 0.20) were significant predictors of higher levels of stigma. Fewer years of education (-4.51, 95% CI = -7.73; -1.28) were additionally significantly associated with higher levels of stigma among caregivers. Factors such as sex, age, school area, years working as a teacher, and caregiver wealth index were not statistically significant factors.

**Conclusion:**

Higher levels of stigma were associated with lower mental health knowledge and higher frequencies of psychological aggression among teachers and caregivers. Additionally, lower caregiver education was associated with higher levels of stigma. This underscores the importance of targeted interventions to enhance mental health awareness and reduce aggressive disciplinary practices. Additionally, the observed associations with caregiver education emphasize the need for tailored approaches that account for demographic variations in stigma.

**Supplementary Information:**

The online version contains supplementary material available at 10.1186/s12889-025-25059-z.

## Background

 The prevalence of child mental disorders is estimated to be between 10 and 20% globally [1, 2], and the World Health Organization (WHO) reports that 80% of those with mental health disorders are from low- and middle-income countries (LMICs) [3]. Uganda has one of the youngest populations in the world, with approximately 55% aged below 18 years, and 44% below 14 years [3]. A recent systematic review investigating mental health disorders in Uganda revealed that 22.9% of children below 18 years old had a mental disorder. More specifically, the prevalence of anxiety and depressive disorders was 14.4% and 22.2%, respectively [1]. The Ugandan government recognized child and adolescent mental health as a critical public health issue in 2017 [4]. Despite the widespread prevalence of mental health problems, recognized public health importance, and acknowledged long-term economic and societal repercussions [5], access to primary health care is limited due to insufficient resources, untrained staff [6], and the pervasive issue of mental health stigma [7]. The stigma surrounding mental health may discourage individuals from seeking help within the healthcare system, resulting in untreated conditions that may worsen over time [7–9].

Teachers and caregivers play crucial roles in identifying, supporting, and referring children with mental health problems [10]. Their attitudes and stigmatizing beliefs can directly influence whether children receive appropriate help and support. Understanding stigma levels among these key adult people in children’s lives is essential because their attitudes can create barriers to help-seeking, delay treatment, and negatively impact children’s mental health outcomes [11]. In 2017, there were only seven psychiatrists in Uganda specialized in child and adolescent psychiatry [12], none of these are at the study site, Mbale. The mental health services are largely provided by specialized nurses and clinical officers; however, they predominantly operate at referral hospitals, regional and national structures within the public health system, and not at lower-level community facilities. The mental health burden increased during and after the Covid-19 pandemic, affecting children in particular, and challenged the mental health structures [13].

The stigma surrounding mental health impacts various aspects of an individual’s life, contributing to social exclusion across key domains such as education, health services, and employment [14, 15]. In addition to this, stigma also largely influences family members associated with the affected individual, community members, healthcare workers attempting to provide help, and treatment options [9]. The sociologist Goffman identified stigma as a discrediting characteristic that reduces a person from a complete, usual person to a tainted one [16]. Goffman was the first to introduce the concept of stigma by association, which is when negative characteristics are attributed to members of the family, friends, and carers of the person being stigmatized. He also identified three other types of stigmas: including stigma due to physical abnormalities, blemishes of character (e.g., mental health issues), and tribal origin (e.g., race, religion) [16]. Goffman emphasizes that the society categorizes individuals and assigns them attributes considered “normal”. When someone displays a characteristic that sets them apart, they may be viewed as flawed or inferior. Thus, the perception of which attributes are discrediting is heavily dependent on cultural context, as some cultures may label certain characteristics more undesirable than others. In Uganda, it is sometimes believed that those with severe mental health problems are bewitched or evil, or that they have done something that makes them deserve suffering [14, 17, 18].

Mental health stigma is a global challenge attributed to a lack of knowledge, negative attitudes, and discriminatory practices [19]. Literature reviews have identified education on mental health and direct social interactions with individuals experiencing mental health problems as effective strategies for mitigating mental health stigma [19, 20]. However, it is noteworthy that these reviews did not specifically address LMICs, where stigma may be heightened due to contextual factors such as poverty coupled with limited education and limited exposure [21]. With an increased emphasis on mental health across diverse platforms, including campaigns and social media [16], it is presumed by some that younger generations exhibit lower levels of mental health stigma compared to their older counterparts [22, 23]. However, some empirical studies indicate that older individuals hold less stigmatized views regarding mental health than younger age groups [22, 24, 25], which could be attributed to more experience with mental health challenges. Additionally, several studies have investigated the link between gender and stigma, with most finding that females are more likely to report more positive attitudes regarding different mental health conditions than males [24, 26–28]. A possible explanation for this may be that females tend to proactively seek and access support services, consequently contributing to diminished levels of stigma.

While no studies to our knowledge have explored the direct link between discipline and stigma, empirical findings consistently show that exposure to violence, including in the context of discipline, can lead to several negative psychological outcomes in childhood and later in life [29]. These findings suggest a potential pathway through which discipline practices may contribute to or reinforce mental health stigma, underscoring the need for further investigation. The relationship between disciplinary practices and mental health stigma may be understood through several potential mechanisms. First, individuals who use harsh discipline may hold more punitive attitudes toward behavioral and emotional difficulties, potentially viewing them as personal failings rather than health issues requiring support [30]. Second, certain disciplinary practices may reflect broader authoritarian beliefs about control and personal responsibility that could extend to attitudes about mental health problems and treatment-seeking [31]. Third, environments characterized by harsh discipline may discourage emotional expression and help-seeking behaviors, potentially reinforcing stigmatizing attitudes toward mental health difficulties [32]. These findings suggest a potential pathway through which discipline practices may contribute to or reinforce mental health stigma, underscoring the need for further investigation.

The use of disciplinary behaviors such as corporal punishment against children, despite being illegal in some countries, including Uganda, remains prevalent in several LMICs [33, 34]. In a randomized controlled trial conducted in 42 primary schools in Uganda, 80% of students reported physical violence by teachers in the past term [35]. Experiencing violence at school can negatively affect students’ mental health [36]. Furthermore, another study found that 95% of secondary school participants (*N* = 663) reported experiencing at least one type of family violence in the past month [37]. Negative consequences of experiencing violence or harsh discipline range from immediate effects such as increased anxiety and behavioral problems to long-term consequences including the development of mental health disorders [38, 39]. Therefore, including a measure of harsh disciplinary violence is not only critical for a comprehensive understanding of child mental health but also for creating more effective interventions.

Other studies have examined relationships of factors such as educational level [40, 41], income [40, 42], and residential area [42, 43] (urban vs. rural) with mental health stigma, with inconsistent results. Regarding educational level, a study of Hispanic women in the U.S. revealed that those with some college education reported significantly less stigma surrounding depression treatment compared to participants with lower education levels [41], while a study from China found no significant differences in stigma across education levels [42]. Regarding income, the same Chinese study found that people with below-median income showed higher devaluation scores toward those with mental illness compared to those with above-median income [42], while research from Indonesia revealed significant differences in stigma levels across various income groups, though the direction of this relationship was not explicitly stated [40]. Lastly, regarding residential area, one study demonstrated that rural residents exhibited higher devaluation scores toward individuals with mental illness compared to urban residents [42], and similarly, an American study found that older adults in isolated rural areas displayed significantly higher levels of both public and self-stigma and lower psychological openness toward mental health treatment than their urban counterparts [43]. These findings demonstrate that sociodemographic factors may influence mental health stigma perceptions, though results vary across different cultural contexts and populations, highlighting the complex relationship between these variables.

A high proportion of children in Uganda (92%) attend primary school [44], making primary school an important arena to not only reach children during their formative years but also to implement task-shifting of mental health initiatives in the educational sector. There have been several child mental health interventions conducted at schools in LMICs, with varying results on long-term effectiveness [45, 46]. A systematic review revealed that interventions addressing depression and anxiety exhibited moderately positive effects [47], urging future interventions to prioritize elements like support from school leaders, and the involvement of teachers and caregivers, in addition to students [47].

This article aims to explore factors associated with mental health stigma among teachers and caregivers of children, with a specific focus on identifying the relationship of stigma with harsh discipline behaviors and mental health knowledge. By examining both of these groups, we will be able to identify factors highly relevant to the stigma children struggling with mental health challenges may experience. To our knowledge, no studies have looked directly at discipline behaviors, such as corporal punishment and psychological aggression, and their association with mental health stigma as the dependent variable. Based on the previously reported literature, we hypothesize a negative association between stigma and mental health knowledge, higher caregiver wealth index, higher age, identifying as a female, education level, living in an urban area, and number of years working as a teacher. Additionally, we hypothesize a positive association between stigmatizing attitudes and the use of corporal discipline and psychological aggression.

## Methods

### Design

The current study used cross-sectional data from the TREAT INTERACT stepped-wedge cluster-randomized trial [48]. The trial intervention is given to school teachers at 18 selected primary schools in Mbale. Data for this study was collected from teachers and caregivers of primary school children at baseline, before the start of the intervention, making this study cross-sectional.

### Setting

The baseline data was collected in September 2023 in Mbale District in eastern Uganda. Mbale serves as a key hub for the transportation of goods and is home to diverse ethnic groups, including the Bamasaba, Banyole, Bagwere, Baganda, Iteso, and Karamojong [49]. The primary languages spoken are Lumasaaba and English. Mbale has a population of approximately 490,000, with approximately 201 primary schools [50]. According to the 2014 census, 53% of the population are under 18 years, and 86% of children aged 6–12 attend primary school. Illiteracy is observed in 29% of individuals who are 18 years or older, and 77% of households are engaged in either crop growing or livestock farming [51]. The district hosts a regional referral hospital with one psychiatric unit having clinical officers (3–4 years of training) specialized in psychiatry and specialized nurses.

Primary schools in Uganda consist of head teachers, teachers, special needs teachers, and senior men and senior women. Senior men and senior women in the Ugandan school system refer to teachers that are involved in providing guidance and counselling [52]. The Ugandan school system is organized as primary school (7 years), secondary school (4 years) corresponding to O-level, and thereafter A-level (2 years), where both O- and A- levels might qualify for further diploma and certificate training. Certificates and Diplomas are 1 to 2-year or more training obtained after O-level or A-level [53].

### Participants and recruitment

Data were collected from 191 primary school teachers and 732 caregivers in Mbale district, Uganda. We used stratified systematic sampling to identify representative primary schools across Mbale District. From an initial pool of 169 educational institutions, we excluded those with existing interventions or scheduled for closure, resulting in 158 eligible schools. This population was then segmented into public (*N* = 107) and private (*N* = 51) institutions, with further classification based on their geographic setting as urban or rural. The selection methodology prioritized creating a sample that accurately represented the diverse educational landscape within the district. We used proportional-to-size sampling techniques within each stratified category to ensure appropriate representation in order to gather comprehensive insights across varying school types and geographical contexts. The final selection comprised 18 primary schools spanning the urban-rural continuum, encompassing both public and private school systems. The selected schools were divided into six cohorts consisting of three schools per cohort, depending on geographical location. Of the 18 selected schools, 13 were public and 5 were private. Regarding geographic distribution, 9 schools were located in urban areas, and 9 in rural settings. All teachers and head teachers were invited to participate in the intervention and research project, and information meetings were held with the head teacher at each school before data collection and training in the TREAT INTERACT intervention began. Schools that agreed to participate in the intervention also agreed to participate in the data collection, although participation in the data collection was a voluntary process agreed to by each individual teacher. After the school had accepted to participate, we used class registration lists to systematically sample child-caregiver pairs proportionate to school size from each school and invited them to participate in the study. Specifically, after obtaining the class lists from the head teacher, all names were combined into one document and were arranged serially before sample selection. An Excel-based software was used to determine the random starting point, sampling interval and the children selected for study participation. The caregivers of these children were then invited to the school. The parents gave separate consent for study participation. The average number of caregivers recruited from each school was 41, while the average number of teachers recruited was 11 (See Table 1).

**Table 1:** Distribution of participants across the 18 schools by location and ownership

	Rural		Urban	
	Private (*N* = 82)	Public (*N* = 335)	Private (*N* = 108)	Public (*N* = 398)
Number of teachers				
Mean (SD)	8.68 (2.51)	12.2 (6.24)	8.96 (2.50)	12.5 (3.71)
Number of caregivers				
Mean (SD)	32.5 (0.50)	39.5 (11.40)	28.0 (7.92)	60.0 (16.30)
Total participants				
Mean (SD)	41.2 (3.01)	51.7 (15.00)	37.0 (5.60)	72.5 (19.50)

### Measures

All scales were piloted and adapted by the research consortium to the Ugandan context. There were no missing data in any of the scales used, and mean scores were rescaled to range from 0 to 100 for ease of interpretation. The complete questionnaires for teachers and caregivers were developed for this trial and can be found in the supplementary materials (Supplementary File 1 and 2).

### Mental health stigma

Stigma surrounding mental health was assessed using a revised version of the Perceived Devaluation-Discrimination (PDD) measure [54–56]. It consists of two categories, one measuring personal stigma (e.g. “Someone who has received mental health treatment is just as trustworthy as the average person”), and the other measuring social distance (e.g. “If I had neighbors with mental illness, I would move out of that neighborhood”). The original scale consists of 12 items [55], but we used 10 items in our study after removing 2 items during piloting due to translation and interpretation difficulties (“I would accept someone who has received mental health treatment as a friend” and “I would think less of a person who has received mental health treatment”). Our final 10-item version uses a 7-point Likert scale (1 = Strongly Disagree – 7 = Strongly Agree). Total scores were calculated by summing the 10 individual item responses, with possible raw scores ranging from 10 to 70. For ease of interpretation, scores were rescaled to range from 0 to 100. Higher scores indicate higher mental health stigma. All analyses treated stigma scores as continuous variables. In the current study, the internal consistency of the scale was α = 0.60.

### Predictor variables

Based on existing literature regarding mental health stigma, we examine the association between stigma and age, sex, education, school area (urban/rural), and number of years working as a teacher for the teacher sample. Education level was categorized into three groups for teachers and caregivers, depending on the number of years it took to complete the degree. In addition to these factors, we also examined the association between stigma and mental health knowledge, discipline, and caregiver wealth index.

#### Mental health knowledge

Teacher and caregiver mental health knowledge was measured with the Mental Health Knowledge Schedule (MAKS) [57]. The scale is intended to measure knowledge and understanding in relation to mental health stigma and consists of one part measuring general knowledge (e.g., “Psychological counseling can be an effective treatment for people with mental health problems”), and a second part measuring the participant’s ability to identify conditions like depression, and schizophrenia as mental illnesses. The internal consistency in the original study was α = 0.65 [57], and a recent study conducted in Tunisia reported α = 0.56 [58]. In the current study, we added conditions that were part of our school intervention curricula (posttraumatic stress disorder, epilepsy, attention deficit hyperactivity disorder, learning and intellectual disabilities, and self-harm and suicide), so the total number of items was 17. Mean scores were calculated on a 5-point Likert scale ranging from 1 = strongly disagree to 5 = strongly agree, with a higher score indicating more knowledge. Internal consistency in the present study was α = 0.74.

#### Discipline

Corporal punishment and psychological aggression were measured by the Dimensions of Discipline Inventory (DDI) [59]. Permission to use this scale was obtained from the original authors. The two subscales constitute 8 items and measure how often a caregiver had used specific discipline behaviors towards a child (e.g., “How often did you spank, slap, smack, or swat a child?”, and “How often did you try to make a child feel ashamed or guilty?). Items were additionally altered to fit the teacher questionnaire. One item (“How often did you hold back affection by acting cold or not giving hugs or kisses?”) was excluded from the current study considering that hugs and kisses are not typical displays of affection in Uganda. Additionally, the DDI originally measures these constructs on a 10-point scale (N = Never, 0 = Not in the past year, but in another year, 1 = 1–2 times in the past year, 2 = 3–5 times in the past year, 3 = 6–9 times in the past year, 4 = Monthly (10 to 14 times in the past year), 5 = A few times a month (2–3 times a month), 6 = Weekly (1–2 times a week), 7 = Several times a week (3–4 times), 8 = Daily (5 or more times a week), and 9 = Two or more times a day). This scale was deemed too comprehensive for the study population after piloting and was adapted to 0 = Never, 1 = Every day, 2 = At least once a week, 3 = At least once a month, and 4 = At least once a year. The authors reported that the internal consistency for corporal punishment was α = 0.80 and α = 0.74 for psychological aggression [59], and a study conducted in Nigeria reported the internal consistency to be α = 0.71 for the whole scale [60]. Calculating score midpoints was one of the methods recommended by the authors for analysis, and this was initially conducted in the current study. However, the Cronbach’s alpha was significantly lower (α = 0.52) compared to using the scaled values (0,1,2,3,4) as is: α = 0.68 for the whole model, and it was therefore decided to use the latter method in data analysis. Alpha for corporal punishment was quite low in the current study (α = 0.46), possibly due to too little variation in item responses. It was therefore excluded from the analyses. Psychological aggression showed a more acceptable alpha (α = 0.60) and was therefore used as the only subscale in analyses. Higher scores indicate more frequent acts of psychological aggression.

#### Caregiver wealth index

A wealth index was created based on the socio-economic status (SES) of the caregiver. An additive scale was used including assets which has previously been used in Uganda [61], and ranged from 0 to 17 (17 = high SES). We did not collect appropriate variables to create a similar index for teachers because our research design focused on occupation-specific variables most relevant to each population. The wealth index included data on various household assets, such as the availability of electricity, radio, bikes, motor vehicles, poultry, and other farm animals.

### Data collection

Data were collected using the offline Open Data Kit (ODK) on smartphones. Experienced research assistants were trained and familiarized with the questionnaire. Interviews were administered by the research assistants for caregivers since many inhabitants in Mbale are not familiar with the use of smartphones and are often more comfortable with speaking. The research assistants read each question to the caregivers, while the interviews for the teachers were self-administered. Research assistants were nearby to assist if needed, and could use a translated version of the questionnaire when necessary.

### Statistical analysis

Descriptive statistics, including means and standard deviations, were stratified by teachers and caregivers. To assess the internal consistency of scales used, the Cronbach alpha function in the ltm package was used. To account for clustering, linear mixed-effects analyses were utilized to examine the differences in mental health stigma, knowledge, and psychological aggression between the teachers and caregivers, and to assess factors associated with mental health stigma for both populations. Prior to analysis, Shapiro-Wilk tests confirmed that while our outcomes showed significant departures from normality (all *p* < 0.001), the W-statistics (0.926–0.966) indicated only modest deviations, supporting the use of linear mixed-effects models with our large sample [62]. Q-Q plots confirmed these findings, showing approximately normal distributions with minor tail deviations (see Supplementary File 3). Variables were included in an unadjusted model and then a multivariable model so that each predictor was controlled for. In the teacher model, we included mental health knowledge, psychological aggression, age, sex, number of years working as a teacher, and school area (urban vs. rural) as fixed effects, or predictors in the model. For caregivers, we included mental health knowledge, psychological aggression, age, sex, wealth index, and school area as fixed effects. Additionally, we conducted a combined analysis including both teachers and caregivers in a single model, with participant type (teacher vs. caregiver) as a fixed effect, to provide direct statistical comparison between groups using variables available for both populations (mental health knowledge, psychological aggression, age, sex, and school area). To account for the variability between different schools, school was included as a random effect in the models. All analyses were conducted in R [63] with the R package nlme [64] for linear mixed effects models.

## Results

Approximately 60% (*N* = 117) of the teachers were females, with a mean age of 40.0 years (SD = 10.6). Teachers had on average worked as a teacher for about 15 years, including 5 years at their current school. Almost 73% of the teachers had a certificate or diploma as the highest completed education (*N* = 139). Due to the large majority of people with a certificate or diploma in this group, educational level was removed from the linear mixed effects analysis as results could be less precise. Headteachers comprised 6.8% of the sampled teachers, while the rest were senior men or senior women (19.9%), special education teachers (7.9%) or regular teachers (65.4%). Approximately 84% of the total sample had not had any previous mental health training (see Table 2).

**Table 2:** Teachers’ sociodemographic characteristics

	Teachers *N* = 191
Age	
Mean (SD)	40.0 (10.6)
Median [Min, Max]	40.0 [21.0, 63.0]
Sex *n* (%)	
Female	117 (61.3)
Male	74 (38.7)
Years working as a teacher	
Mean (SD)	15.4 (9.5)
Median [Min, Max]	13.0 [1.0, 38.0]
Years working in current school	
Mean (SD)	5.2 (4.4)
Median [Min, Max]	4.0 [1.0, 26.0]
Level of education *n* (%)	
O-level/A-level	25 (13.1)
*Vocational training institutions*	
*Certificate/Diploma*	139 (72.8)
University training	
*Degree*,* Master*,* Post-graduate*, PhD*	27 (14.1)
Previous mental health training *n* (%)	
After teacher’s training	12 (6.3)
During teacher’s training	19 (9.9)
None	160 (83.8)
Position *n* (%)	
Headteacher	13 (6.8)
Teacher	125 (65.4)
Senior man teacher/Senior women teacher	38 (19.9)
Special Needs Teacher	15 (7.9)

*Continuous variables are provided as mean (SD) and medians [min*,* max]*,* and categorical as n (percentages)*

*PhD included 1 person, Post-graduate: 2, Master: 24. The rest had lower degrees

As for the caregivers, the total sample included in the study was 732. The mean age was 40.7 years (SD = 13.0), 72% were female, and 56% had their children enrolled in an urban school. More than half of the caregivers were mothers to the child participating in the study, and 80% were married. Caregivers had a median of 5 children, ranging from 0 up to 36 (it is common for some parents to have several children, as many still follow the cultural tradition of polygamy, and some caregivers take caregiver responsibilities for orphaned children or children with other needs). More than half (58%) of the caregivers had completed either lower secondary or both lower and upper secondary school education. The caregivers had an average income of approximately 59 USD per month, and 67% were peasants or farmers (see Table 3).

**Table 3:** Caregivers’ sociodemographic characteristics

	Caregivers (*N* = 732)
Age	
Mean (SD)	40.7 (13.0)
Median [Min, Max]	38.0 [15.0, 88.0]
Sex *n* (%)	
Female	524 (71.6)
Male	208 (28.4)
Area *n* (%)	
Rural	322 (44.0)
Urban	410 (56.0)
Marital status *n* (%)	
Married	573 (78.3)
Divorced/Separated	22 (3.0)
Co-habiting	3 (0.4)
Single	66 (9.0)
Widowed	68 (9.3)
Who are you in relation to the child *n* (%)	
Mother	401 (54.8)
Father	150 (20.5)
Grandma/pa	87 (11.9)
Other	94 (12.8)
No. of children	
Median [Min, Max]	5.0 [0, 36.0]
Education *n* (%)	
0 to 7 years of schooling	
*(No education*,* primary school)*	309 (42.2)
8 to 11 years of schooling	
*(Lower secondary school S1-S4)*	233 (31.8)
12 + years of schooling	
*(Upper secondary and above)*	190 (26.0)
Main source of income *n* (%)	
Peasant/farmer	487 (66.5)
Commercial farmer	15 (2.0)
Other business activities	55 (7.5)
Student	9 (1.2)
Other	166 (22.7)
Wealth Index (range 0–17)	
Mean (SD)	3.2 (2.6)
Median [Min, Max]	2.0 [1.0,15.0]
Monthly income (USD)	
Mean (SD)	58.7 (182.84)
Median [Min, Max]	27.3 [0, 3820.5]

*Continuous variables provided as mean (SD) and medians [min*,* max] and categorical as n (percentages)*

### Stigma scores, mental health knowledge and discipline

**Fig. 1:**
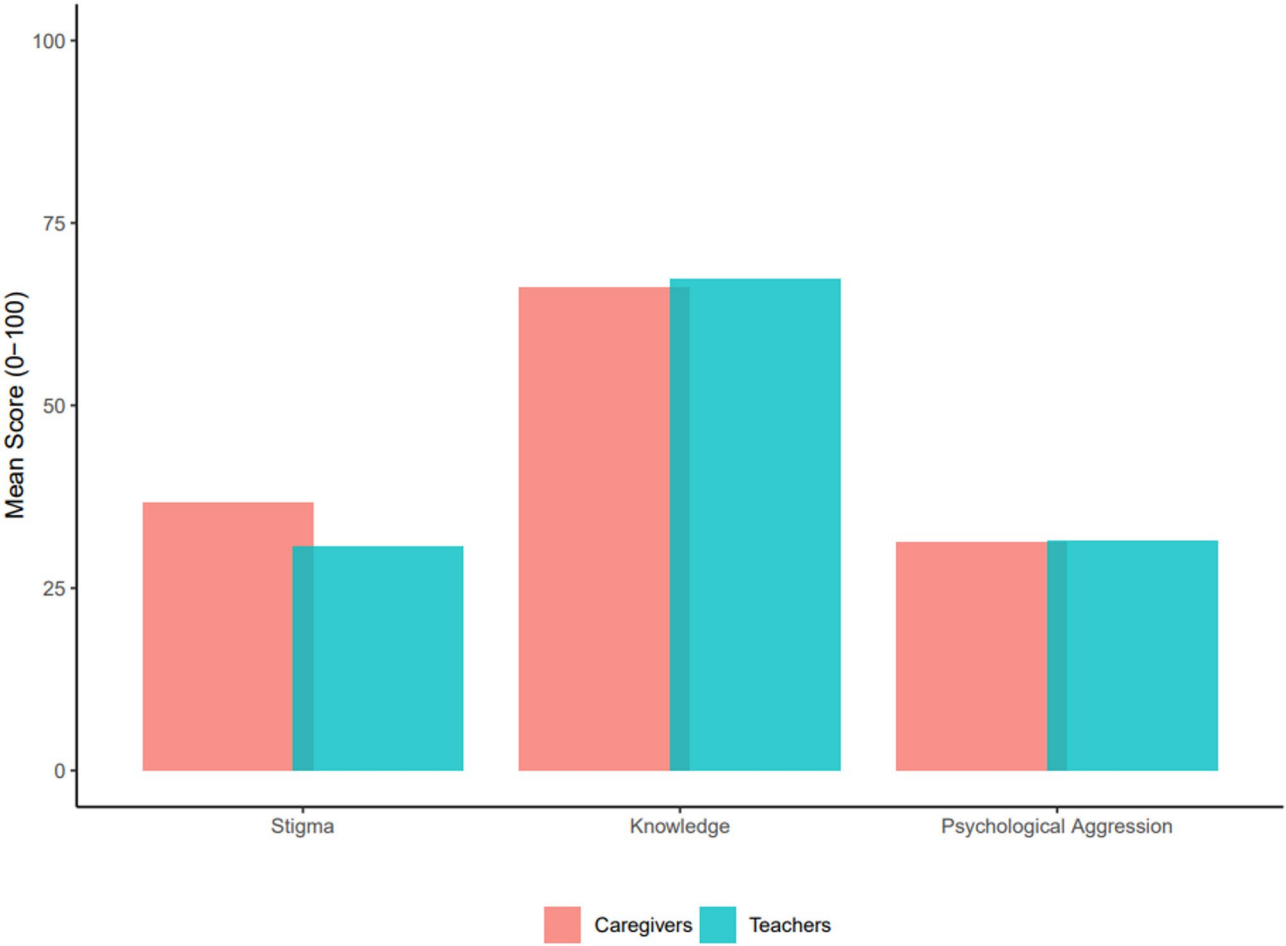
Mean scores for mental health stigma, knowledge, and psychological aggression by group. All scales range from 0–100, with higher scores indicating more of the respective construct

On the mental health stigma scale (PDD), teachers scored an average of 30.7 (SD = 14.0), while caregivers had a mean score of 36.8 (SD = 16.9). Higher scores indicate more stigma, demonstrating that caregivers reported more stigmatizing attitudes related to mental health on average as compared to the teachers (Fig. 1). The difference between the two groups was statistically significant (−5.93, 95% CI = −8.54; −3.33). An insignificant difference (1.08, 95% CI = −1.20; 3.38) was found between teachers’ and caregivers’ knowledge of mental health (M = 67.3, SD = 13.0 and M = 66.2, SD = 14.7, respectively). As for psychological aggression, teachers had a mean score of 31.5 (SD = 26.8) and caregivers 31.3 (SD = 24.8), and this difference was not significant (0.26, 95% CI = −3.75; 4.26). Adjusting for differences in age and sex between the two populations revealed similar results (see Table 4). This indicates that teachers had similar mental health knowledge and displayed similar acts of psychological aggression as caregivers. There was a significant difference in the sex distribution between caregiver and teacher respondents (*p* = 0.005), where 71.6% of caregivers were female, and 61.3% of teachers were female. There were no significant differences in age between the two populations (*p* = 0.962).

**Table 4:** Teachers’ and caregivers’ mean values (SD) and differences for mental health stigma, mental health knowledge, and psychological aggression

	Mean (SD)	Unadjusted Test of difference – (95%CI) *p*	Adjusted* Test of difference – (95%CI) *p*
Mental health stigma			
Teachers	30.7 (14.0)	−5.93 (−8.54; −3.33), *p* < 0.001	−6.00 (−8.62; −3.39), *p* < 0.001
Caregivers	36.8 (16.9)		
Mental health knowledge			
Teachers	67.3 (13.0)	1.08 (−1.20; 3.38), *p* = 0.352	1.25 (−1.06; 3.55), *p* = 0.289
Caregivers	66.2 (14.7)		
Psychological Aggression			
Teachers	31.5 (26.8)	0.26 (−3.75; 4.26), *p* = 0.900	0.66 (−3.37; 4.68), *p* = 0.749
Caregivers	31.3 (24.9)		

All scales range from 0 to 100.

*Adjusted for sex and age

### Factors associated with teacher mental health stigma

Multivariable linear mixed effects analysis revealed that both less mental health knowledge (−0.37, 95% CI = −0.52; −0.23) and more psychological aggression (0.14, 95% CI = 0.07; 0.22) were significant predictors of higher levels of mental health stigma among the teachers (see Table 5). Sex, age, number of years working as a teacher, and school area were not significantly related to stigma in the adjusted, multivariable analysis. 

**Table 5:** Linear mixed effects analysis showing the relation between teachers’ mental health stigma*and predictor variables

	Unadjusted model			Adjusted model		
*Predictors*	*Estimates*	*95% CI*	*p*	*Estimates*	*95% CI*	*p*
Sex [Female = 1]	0.75	−3.32; 4.81	0.717	1.67	−2.14; 5.49	0.388
Age, years	0.14	−0.05; 0.34	0.150	0.20	−0.19; 0.58	0.316
No. of years working	0.10	−0.12; 0.32	0.377	−0.10	−0.53; 0.34	0.662
School area [Urban]	−0.98	−6.80; 4.85	0.727	−1.97	−7.46; 3.53	0.459
Mental health knowledge*	−0.33	−0.48; −0.18	< 0.001	−0.37	−0.52; −0.23	< 0.001
Psychological aggression*	0.11	0.04; 0.19	0.003	0.14	0.07; 0.22	< 0.001

*Scales from 0–100: a unit increase in exposure scale and effects on the outcome stigma scale (0–100)

Factors associated with caregiver mental health stigma

Results from the caregiver model align with those of the teacher model, indicating that lower mental health knowledge was associated with more stigma (−0.25, 95% CI = −0.33; −0.17), and psychological aggression was slightly positively linked to stigma (0.15, 95% CI = 0.10; 0.20). Caregivers with more than 11 years of education also reported significantly lower mental health stigma compared to those with no education or only primary school education (−4.51, 95% CI = −7.73; −1.28). Other factors, such as sex, age, school area, and wealth index were not significantly related to mental health stigma among caregivers. The unadjusted model did not reveal significant associations differing from the adjusted, multivariable model (see Table 6).

**Table 6:** Linear mixed-effects analysis showing the correlation between caregiver’s mental health stigma* and predictor variables

Unadjusted model				Adjusted model		
*Predictors*	*Estimates*	*CI*	*p*	*Estimates*	*CI*	*p*
Sex [Female = 1]	−0.46	−3.21; 2.29	0.776	−2.01	−4.81; 0.79	0.160
Age	−0.05	−0.15; −0.04	0.269	−0.08	−0.18; 0.01	0.096
Wealth Index	−0.05	−0.54; 0.44	0.842	−0.00	−0.50; 0.49	0.987
Education [8–11 y]	−0.27	−3.13; 2.59	0.852	−0.99	−3.81; 1.84	0.492
Education [12 + y]	−3.93	−7.07; −0.80	0.014	−4.51	−7.73; −1.28	0.006
School area [Urban]	0.00	−4.25; 4.25	0.999	0.72	−3.34; 4.77	0.713
Mental health knowledge*	−0.21	−0.29; −0.13	< 0.001	−0.25	−0.33; −0.17	< 0.001
Psychological aggression*	0.13	0.08; 0.18	< 0.001	0.15	0.10; 0.20	< 0.001

*Scales from 0 to 100: results show the effect of a unit increase from the respective prediction scales (0–100) on the outcome scale (0–100)

Additionally, the combined model analysis confirmed significant differences between teachers and caregivers while controlling for shared variables. Teachers demonstrated significantly lower mental health stigma than caregivers (−5.78, 95% CI = −8.28; −3.27, *p* < 0.001). Consistent with the separate analyses, mental health knowledge was associated with lower stigma (−0.27, 95% CI = −0.34; −0.19, *p* < 0.001) and psychological aggression was associated with higher stigma (0.14, 95% CI = 0.10; 0.18, *p* < 0.001) across both groups.

## Discussion

The goal of this study was to examine factors related to mental health stigma among caregivers of primary school children and their teachers. Mental health stigma is one of the key barriers to seeking mental health treatment [11]. Examining the factors associated with stigma is crucial for understanding specific areas to target in efforts to reduce stigma and its impact. In our study, the average stigma scores were 30.7 (SD = 14.0) for teachers and 36.8 (SD = 16.9) for caregivers on a scale from 0 to 100. Higher mental health knowledge, and higher level of education were significant predictors of lower levels of stigma, which is similar to findings from other studies [24, 40, 65].

### Mental health knowledge

The results from the current study are in line with findings from previous studies regarding mental health knowledge [19, 20, 42], suggesting that higher mental health knowledge is associated with lower mental health stigma. Some longitudinal studies have attempted to address stigma by increasing mental health literacy. For instance, a study conducted in Germany which examined the impact of labelling a condition as a mental illness on public attitudes towards individuals with depression and schizophrenia, found that labelling had a negative effect on stigma towards those with schizophrenia, but not those with depression. The authors suggested that this may stem from the stereotype associating individuals with schizophrenia as more threatening than those with depression and emphasized the need to distinguish between various mental illnesses when addressing stigma. However, that study did not control for participants’ pre-existing knowledge about mental health [66]. In contrast, another study conducted among a representative sample of the Swiss residents found that participants with higher mental health literacy exhibited more negative attitudes and a preference for social distance from people with mental illness, indicating that higher mental health literacy does not always signify lower levels of stigma [67]. Similar results from another study indicated that enhancing mental health literacy within a highly educated population might not yield the intended outcome of less stigma, and that when addressing stigma, educational campaigns should consider the distinctive traits of their target population [68]. A possible explanation for the variation in findings could be found in systematic reviews examining interventions targeting mental health stigma in LMICs. Longer training interventions (more than one day) with role-plays and discussion of case studies were more likely to produce significant changes in stigma compared to brief training interventions [69]. Another potential explanation for the variation in findings across studies could be the cultural adaptation of the training and of the measurement tools.

It is also important to note that the internal consistency of the stigma scale was quite low (α = 0.60), suggesting that items within the scale may not consistently be measuring the same underlying construct. The perception, experience, and management of stigma is complex and deeply culturally sensitive, which is why using tools developed in the Global North to measure stigma is problematic. The process of understanding what is considered normal varies greatly in different cultures, and the language in which we speak also heavily influences the view of mental health disorders [70]. Tools developed in the Global North contexts often carry assumptions about norms and behaviors that may not translate conceptually to other cultures with different understandings of mental health.

### Psychological aggression

Teacher and caregiver acts of psychological aggression were positively related with mental health stigma. Previous studies conducted in LMICs have showed that using various forms of discipline at home and at school is considered normal [33, 71], and often thought of as a necessary strategy for ensuring good behavior [72]. The previously mentioned statistics regarding the prevalence of school and family violence [37, 73] alongside the present results underscore the relevance of examining types of discipline behaviors in relation to mental health stigma. It is worth noting that most studies examining these factors focus specifically on corporal punishment, which could not be investigated in the current study due to the low internal consistency of the subscale. Despite the absence of prior studies examining this, an intervention study aiming to increase mental health literacy found decreases in both mental health stigma and harsh discipline behaviors after program implementation [74], suggesting an association between literacy, stigma and violence. An intervention study among school staff in Uganda [35] provided new knowledge on alternative non-violent discipline through facilitation and activity guides, encouraging empathy and setting school-wide goals demonstrated positive outcomes post-implementation. They found that prevalence of physical violence was lower in interventions schools than in control schools (31% vs. 48.7%). These findings imply that enhancing knowledge about the effects of discipline behaviors may contribute to an overall increase in mental health literacy, subsequently reducing stigma and reducing violence. To further investigate this concept, data from repeated measures should be used. This approach allows for a comprehensive examination of the effects not only post-intervention but also across multiple time points throughout the intervention period.

### Education

Caregivers with over 11 years of education (upper secondary school and above) reported significantly lower mental health stigma than those with lower education (no education/primary school). Although previous studies have not always found consistent results regarding the role of education level [40], there is a body of evidence pointing to the links between higher education and less discriminatory attitudes [65, 75].

### Other predictors

There were no significant results regarding sex and mental health stigma, despite previous studies finding that females usually exhibit less stigmatized attitudes [24, 28]. Similarly, factors such as years of working as a teacher, caregiver wealth index, and school area did not produce significant findings. Future interventions should consider both literacy and different forms of discipline when designing and implementing programs targeting mental health stigma.

### Limitations and future directions

The current study used baseline data from a stepped-wedge trial with data from 18 primary schools in Mbale, Uganda to investigate factors associated with mental health stigma among caregivers and teachers. Therefore, this study had some limitations including the inability to establish causal relationships for our findings. It is also unclear how the constructs measured in the study change over time with increased knowledge. Future studies should focus on examining these factors over time using a repeated measures design. Questionnaire data were sampled through interviews with caregivers. Although this method is widely used in LMICs, it carries the potential risk of introducing bias, particularly regarding less reliable responses from participants. It is important to emphasize the need for trained data collectors who have experience asking participants about these sensitive topics. Socio-economy was measured using an additive scale, which is commonly used due to the ease of data collection and reliability of data. However, this approach might not fully represent the complete picture of wealth, as it does not adequately consider factors like ownership, access, and distribution. As mentioned earlier, the measure for mental health stigma (PDD) [55] had a medium level internal consistency in this setting. The low internal consistency for measuring corporal punishment was also a limitation and resulted in the removal of the scale in the analysis. This highlights the need for future design and/or adaptation of tools relevant for this context. That said, to our knowledge, this is the first study to directly investigate the relationship between psychological aggression towards children and mental health stigma and we call for urgent public health attention in this regard.

## Conclusion

The current study revealed that mental health knowledge, psychological aggression, and caregiver education were significant factors related to mental health stigma in a sample of 191 teachers and 732 caregivers. Reducing mental health stigma stands as a crucial step in overcoming barriers to seeking help. Decreasing stigma could lead to significant changes in the number who seek help for mental health issues during the early stages, preventing the escalation of the mental health challenge. Ultimately, lower levels of stigma may contribute to a lower long-term prevalence of individuals facing mental health challenges. Although this study focused on mental health stigma in two distinct groups - teachers and caregivers - there is a notable consistency in the models concerning mental health knowledge and psychologically aggressive forms of discipline. In both samples, a consistent pattern emerged: a negative relation between mental health knowledge and stigma, and a positive association between psychological aggression and stigma. This suggests that these factors remain consistent across various populations, strengthening their importance in the effort to reduce mental health stigma. Consequently, interventions directed at these specific constructs could be instrumental in reducing stigma and reducing psychological aggression.

## Supplementary Information

Supplementary Material 1.

Supplementary Material 2.

Supplementary Material 3.

## Data Availability

The datasets used and analyzed during the current study are available from the corresponding author upon reasonable request.
