# Supplementary material for: Factors associated with mental health stigma among teachers and caregivers of primary school children in Uganda
Source: BMC Public Health. 2025 Nov 4;25:3771. doi: 10.1186/s12889-025-25059-z (PMC12584251; doi:10.1186/s12889-025-25059-z)
Supplement: Supplementary file 1 — Supplementary Material 1. [file 12889_2025_25059_MOESM1_ESM.doc]

| **DOCUMENT** | **DATA DICTIONARY** |
| --- | --- |
| **STUDY TITLE** | **TREAT INTERACT: IMPLEMENTING A USER INVOLVED**  **EDUCATION- AND HEALTH SYSTEM INTERACTIVE TASK-**  **SHIFTING APPROACH FOR CHILD MENTAL HEALTH**  **PROMOTION IN UGANDA.** |
| **STUDY GROUP** | **CARETAKERS** |
| **INSTITUTIONS** | **MAKERERE UNIVERSITY SCHOOL OF PUBLIC HEALTH, NORWEGIAN CENTER FOR VIOLENCE AND TRAUMATIC STRESS STUDIES, UNIVERSITY OF BERGEN, THE NORWEGIAN UNIVERSITY OF SCIENCE AND TECHNOLOGY AND THE NORWEGIAN INSTITUTE FOR OF PUBLIC HEALTH** |
| **BIOSTATISTICIAN** | **KALIBBALA DENNIS**  [**kalibbaladennis@gmail.com**](mailto:kalibbaladennis@gmail.com) **+256 (0) 777666152 /750631315** |
| **DATA MANAGER** | **OLET STEPHEN CHARLES**  [**stecho36@gmail.com**](mailto:stecho36@gmail.com) **+256 (0) 777824203, 0757433711** |
| **MAIN INVESTIGATORS** | **1. JULIET NDIMWIBO BABIRYE**  **2. INGUNN ENGEBRETSEN**  **3. NORBERT SKOKAUSKAS**  **4. NORA BRAATHU**  **5. ESTHER KISAKYE**  **6. MUKISA MARJORIE KABATOORO**  **7. JOYCE SSERUNJOGI NALUGYA**  **8. HARRIET ABER**  **9. VILDE SKYLSTAD**  **10. MELF-JAKOB KÜHL**  **11. TORE WENTZEL-LARSEN**  **12. HARALD BÆKKELUND**  **13. ANE-MARTHE SOLHEIM SKAR** |
| **DATE** | **6^TH^ SEPTEMBER 2023** |

| **S/N** | **VARIABLES** | **DESCRIPTION** | **RESPONSE OPTIONS** | **TYPE OF VARIABLE** |
| --- | --- | --- | --- | --- |
| Unique Identifiers | | | | |
| 1 | IDschool | ID number of school of the child | Numeric | Discrete |
| 2 | Schoolname | Name of school | Text | String |
| 3 | IDchild | ID number of child participating in the study | Numeric | Discrete |
| 4 | IDcaretaker | ID number of caretaker (participant) | Numeric | Discrete |
| Demographics | | | | |
| General | | | | |
| 5 | Date | Today's Date | Date | Ordinal |
| 6 | Demographics1 | Urban/rural? | 1= urban  2= rural  3= semi-urban | Nominal |
| 7 | Demographics2 | What is the primary language in the household? | 1=Lumasaaba  2=Ateso  3=Lugwere  4=Luganda  5=English  6=Other | Nominal |
| 8 | Specifydemographics2 | Specify | Text | String |
| Household composition | | | | |
| 9 | Household1 | What is your marital status? | 1=Single  2=Married  3=Co-habiting  4=Widowed  5= Divorced/Separated | Nominal |
| 10 | Household2 | Who are you in relation to the child/children participating in this study? | 1=Mother  2=Father  3=Uncle/aunt  4=Grandma/pa  5=Sibling  6= Cousin  7=Employed at orphanage/child institution  8=Others | Nominal |
| 11 | Specifyhousehold2 | Specify | Text | String |
| 12 | Livedchild | Have you lived with the child their whole life? | 0=No  1=Yes | Binary |
| 13 | Ifnotlivedchild | How long have you been living with the child? | Numeric | Continuous |
| 14 | Household4 | How old are you? | Numeric | Continuous |
| 15 | Household5 | What is your sex? | 0 = Male  1 = Female | Binary |
| 16 | Household6 | How many children do you have? | Numeric | Continuous |
| Caregiver education status | | | | |
| 17 | Household7 | Have you ever attended school? | 0 = no  1= yes | Binary |
| 18 | Household8 | What is your highest level of education? | 0= preschool  1=P1 (Primary)  2=P2 (Primary)  3=P3 (Primary)  4=P4 (Primary)  5=P5 (Primary)  6=P6 (Primary)  7=P7 (Primary)  8=S1 (Senior)  9=S2 (Senior)  10=S3 (Senior)  11=S4 O-level (Senior)  12=S5 (Senior)  13=S6 A-level (Senior)  14=Certificate: 1 Year  15=Certificate: 2 Years  16=Degree/Bachelor  17=Education higher than bachelor | Ordinal |
| 19 | Household9 | Can you read? | 0 = no  1= yes | Binary |
| 20 | Household10 | Can you write? | 0 = no  1= yes | Binary |
| Economic status | | | | |
| 21 | Economicstatus12 | Do you have a regular or irregular salary? | 1= Regular salary work  2= Irregular salary | Nominal |
| 22 | Economicstatus13 | What is your main occupation/source of income? | 1=No income generating activities  2=Peasant/farmer  3=Petty trader  4=Commercial farmer  5= Brewing or selling alcohol  6=Shop keeper  7=Student  8=Other | Nominal |
| 23 | Specifyeconomicstatus13 | Specify | Text | String |
| Partner | | | | |
| 24 | Economicstatus14 | Does your partner have a regular or irregular source of income? | 1= Regular salary  2= Irregular salary | Nominal |
| 25 | Economicstatus15 | What is your partner's main occupation/source of income? | 1=No income generating activities  2=Peasant/farmer  3=Petty trader  4=Commercial farmer  5= Brewing or selling alcohol  6=Shop keeper  7=Student  8=Other | Nominal |
| 25 | Specifyeconomicstatus15 | Specify | Text | String |
| 27 | Economicstatus16 | Does your partner take part in the family income generating activity? | 0=no  1=yes | Binary |
| 28 | Economicstatus17 | Does your partner earn money? | 0=no  1=yes | Binary |
| 29 | Economicstatus18 | What is the household monthly income? | Numeric | Continuous |
| 30 | Economicstatus19 | Is the place you live in free, rented or owned by your household members? | 1=Free  2=Rent  3=Own  9=DNK | Nominal |
| 31 | Economicstatus20 | Does someone in your household own land? // Do you or your partner own land? | 0=No  1=Yes  9=DNK | Nominal |
| 32 | Economicstatus21 | How big is the land? | 1=Less than 1 acre  2=Between 1-5 acre  3= More than 5 acres | Ordinal |
| 33 | Economicstatus22 | Did you or other members of your household have to borrow money or sell assets in order to pay for rent, health costs, school fees, or any other costs during the last 12 months? | 0=no  1=yes | Binary |
| 34 | Economicstatus23 | What was the main reason? | 1=pay for rent  2=health costs  3=school fees  4=Other | Nominal |
| 34 | Specifyeconomicstatus23 | Specify | Text | Text |
| 36 | Economicstatus24 | How much debt (loan) do you have for the current year? | Numeric | Continuous |
| 37 | Economicstatus25 | Do you have electricity in the house you live in? | 0=no  1=yes | Binary |
| 38 | Economicstatus26 | Do you have any of the following in your household? (only items that work) | 1= cupboard  2=Mobile phone (ring / SMS)/telephone (not smartphone)  3=Smartphone (internet option)  4=Radio  5=TV  6=Laptop/Computer  7=Refrigerator  8=Bicycle  9=Motor cycle/scooter  10=Car/truck  11=Bed with mattress  12=insecticide treated mosquito net  13=Dining table | Nominal |
| 39 | Usedany | Have you used any of the following the last month | 1=internet  2=TV  3=Radio  4=Newspaper | Nominal |
| 40 | Economicstatus27 | What is the main source of drinking water in your household now? | 1=Pond, river or stream  2=Unprotected natural spring  3=Protected natural spring  4=Rain water  5=Open or unprotected well  6=Covered well  7=Borehole  8=Public tap  9=Piped into yard/plot  10=Piped into dwelling  11=Bottled water | Nominal |
| 41 | Economicstatus28 | What is the main cooking fuel used in your household now? | 1=Wood  2=Charcoal  3=Paraffin/kerosene  4=Gas  5=Electricity | Nominal |
| 42 | Economicstatus29 | Where do you go to the toilet? | 1=No/In the open  2=In a bucket  3=In a pit latrine  4=In a VIP latrine  5=Flush toilet | Nominal |
| 43 | Economicstatus30 | Do you share this/your toilet with any neighbouring households? | 0=No  1=Yes | Binary |
|  | Livestock | How many of the following do you have? |  |  |
| 44 | Cows | Cows | Numeric | Continuous |
| 45 | Goats | Goats | Numeric | Continuous |
| 46 | Pigs | Pigs | Numeric | Continuous |
| 47 | Poultry | Poultry | Numeric | Continuous |
| 48 | Economicstatus31 | Main material of the floor | 1=Soil/earth/dung  2=Cement  3=Tiles  4=Rudimentary wooden  5=Finished wooden  6=Carpet/Vinyl  7=Other | Nominal |
| 49 | Specifyeconomicstatus31 | Specify | Text | String |
| 50 | Economicstatus32 | Main material of the roof | 1=Grass thatched  2=Iron sheets  3=Tiles  4=Concrete  5=Wood  6=Other | Nominal |
| 51 | Specifyeconomicstatus32 | Specify | Text | String |
| 52 | Economicstatus33 | Main material of the walls | 1=Mud and pole  2=Wood  3=Bricks without mortar  4=Bricks with mortar  5=Plastered walls  6=Iron sheets  7=Other | Nominal |
| 53 | Specifyeconomicstatus33 | Specify | Text | String |
| 54 | Economicstatus34 | Main material of windows | 1=No material  2=Plastic  3=Wood  4=Glass  5=Other | Nominal |
| 55 | Specifyeconomicstatus34 | Specify | Text | String |
| 56 | Economicstatus35 | Main material of doors | 1=No doors  2=Only outer doors  3=Outer and inner doors  4=Other | Nominal |
| 57 | Specifyeconomicstatus35 | Specify | Text | String |
| 58 | Economicstatus36 | The data collector ticks off the type of house the child lives in | 1=Shack  2=Traditional hut  3=Semi-permanent house  4=Permanent house  5=Other | Nominal |
| 59 | Specifyeconomicstatus36 | Specify | Text | String |
| About help-seeking | | | | |
| 60 | Speak | At any point during the past 3 months, did you ever speak to a health professional about any mental health problem or concern? | 1= I did not have any mental health problem or concern  2=I am waiting to see a health professional about a mental health problem or concern  3=I spoke to a health professional about a mental health problem or concern  4=I decided not to speak to a health professional although I am concerned about my mental health | Nominal |
|  | | | | |
| 61 | Who1 | My mother or father (or equivalent) | 1=Did not feel the need to ask for help  2=Wanted to but did not ask for help  3=Asked for help | Nominal |
| 62 | Who2 | A sibling (brother, sister, step sibling, etc.) | 1=Did not feel the need to ask for help  2=Wanted to but did not ask for help  3=Asked for help | Nominal |
| 63 | Who3 | Another relative (or equivalent) | 1=Did not feel the need to ask for help  2=Wanted to but did not ask for help  3=Asked for help | Nominal |
| 64 | Who4 | A close friend | 1=Did not feel the need to ask for help  2=Wanted to but did not ask for help  3=Asked for help | Nominal |
| 65 | Who5 | A work colleague | 1=Did not feel the need to ask for help  2=Wanted to but did not ask for help  3=Asked for help | Nominal |
| 66 | Who6 | My minister, priest, rabbi, imam (or some other spiritual or religious leader) | 1=Did not feel the need to ask for help  2=Wanted to but did not ask for help  3=Asked for help | Nominal |
| 67 | Who7 | Traditional healer | 1=Did not feel the need to ask for help  2=Wanted to but did not ask for help  3=Asked for help | Nominal |
| 68 | Who8 | My regular family health professional (e.g., a physician or a nurse) | 1=Did not feel the need to ask for help  2=Wanted to but did not ask for help  3=Asked for help | Nominal |
| 69 | Who9 | A mental health professional (like a counselor, psychologist, psychiatrist, or mental health nurse) | 1=Did not feel the need to ask for help  2=Wanted to but did not ask for help  3=Asked for help | Nominal |
| 70 | Who10 | A person not identified above | 1=Did not feel the need to ask for help  2=Wanted to but did not ask for help  3=Asked for help | Nominal |
| Attitudes about Mental Health | | | | |
| 71 | Attitude1 | A person who has received mental health treatment is just as intelligent as everyone else | 1=Strongly Disagree  2=Disagree  3=Disagree a little  4=Not sure  5=Agree a little  6=Agree  7=Strongly Agree | Ordinal |
| 72 | Attitude2 | Someone who has received mental health treatment is just as trustworthy as everyone else | 1=Strongly Disagree  2=Disagree  3=Disagree a little  4=Not sure  5=Agree a little  6=Agree  7=Strongly Agree | Ordinal |
| 73 | Attitude3 | It is acceptable that someone who has fully recovered from a mental illness can work as a teacher of young children in a public school. | 1=Strongly Disagree  2=Disagree  3=Disagree a little  4=Not sure  5=Agree a little  6=Agree  7=Strongly Agree | Ordinal |
| 74 | Attitude4 | Receiving mental health treatment is a sign of personal failure or weakness. | 1=Strongly Disagree  2=Disagree  3=Disagree a little  4=Not sure  5=Agree a little  6=Agree  7=Strongly Agree | Ordinal |
| 75 | Attitude5 | People with severe mental illness can have good quality of life, regardless of treatment. | 1=Strongly Disagree  2=Disagree  3=Disagree a little  4=Not sure  5=Agree a little  6=Agree  7=Strongly Agree | Ordinal |
| 76 | Attitude6 | People with mental illness are to blame for their own condition. | 1=Strongly Disagree  2=Disagree  3=Disagree a little  4=Not sure  5=Agree a little  6=Agree  7=Strongly Agree | Ordinal |
| 77 | Attitude7 | If my colleague told me he or she had a mental illness, I would still want to work with him or her. | 1=Strongly Disagree  2=Disagree  3=Disagree a little  4=Not sure  5=Agree a little  6=Agree  7=Strongly Agree | Ordinal |
| 78 | Attitude8 | If I had neighbors with mental illness, I would move out of that neighborhood. | 1=Strongly Disagree  2=Disagree  3=Disagree a little  4=Not sure  5=Agree a little  6=Agree  7=Strongly Agree | Ordinal |
| 79 | Attitude9 | If a person who had fully recovered from mental illness asked me for a letter of support to get employment, I would provide a reference. | 1=Strongly Disagree  2=Disagree  3=Disagree a little  4=Not sure  5=Agree a little  6=Agree  7=Strongly Agree | Ordinal |
| 80 | Attitude10 | If I had a mental illness, I would not admit this to any of my friends for fear of being treated differently. | 1=Strongly Disagree  2=Disagree  3=Disagree a little  4=Not sure  5=Agree a little  6=Agree  7=Strongly Agree | Ordinal |
| Mental Health Knowledge | | | | |
| 81 | Knowledge1 | Most people with mental health problems want to work | 1=Disagree strongly  2=Disagree slightly  3=Neither disagree nor agree  4=Don’t know  5=Agree slightly  6=Agree strongly | Ordinal |
| 82 | Knowledge2 | If a friend had a mental health problem, I would advise him or her to get professional help | 1=Disagree strongly  2=Disagree slightly  3=Neither disagree nor agree  4=Don’t know  5=Agree slightly  6=Agree strongly | Ordinal |
| 83 | Knowledge3 | Medication can be an effective treatment for people with mental health problems | 1=Disagree strongly  2=Disagree slightly  3=Neither disagree nor agree  4=Don’t know  5=Agree slightly  6=Agree strongly | Ordinal |
| 84 | Knowledge4 | Psychological counselling can be an effective treatment for people with mental health problems | 1=Disagree strongly  2=Disagree slightly  3=Neither disagree nor agree  4=Don’t know  5=Agree slightly  6=Agree strongly | Ordinal |
| 85 | Knowledge5 | People with severe mental health problems can fully recover, with treatment | 1=Disagree strongly  2=Disagree slightly  3=Neither disagree nor agree  4=Don’t know  5=Agree slightly  6=Agree strongly | Ordinal |
| 86 | Knowledge6 | People with severe mental health problems can fully recover, without treatment | 1=Disagree strongly  2=Disagree slightly  3=Neither disagree nor agree  4=Don’t know  5=Agree slightly  6=Agree strongly | Ordinal |
| 87 | Knowledge7 | Most people with mental health problems go to a healthcare professional to get help | 1=Disagree strongly  2=Disagree slightly  3=Neither disagree nor agree  4=Don’t know  5=Agree slightly  6=Agree strongly | Ordinal |
| Type of mental illness | | | | |
| 88 | Knowledge8 | Depression | 1=Disagree strongly  2=Disagree slightly  3=Neither disagree nor agree  4=Don’t know  5=Agree slightly  6=Agree strongly | Ordinal |
| 89 | Knowledge9 | Stress | 1=Disagree strongly  2=Disagree slightly  3=Neither disagree nor agree  4=Don’t know  5=Agree slightly  6=Agree strongly | Ordinal |
| 90 | Knowledge10 | Schizophrenia | 1=Disagree strongly  2=Disagree slightly  3=Neither disagree nor agree  4=Don’t know  5=Agree slightly  6=Agree strongly | Ordinal |
| 91 | Knowledge11 | Bipolar disorder (manic depression) | 1=Disagree strongly  2=Disagree slightly  3=Neither disagree nor agree  4=Don’t know  5=Agree slightly  6=Agree strongly | Ordinal |
| 92 | Knowledge12 | Alcohol and other drug use disorders | 1=Disagree strongly  2=Disagree slightly  3=Neither disagree nor agree  4=Don’t know  5=Agree slightly  6=Agree strongly | Ordinal |
| 93 | Knowledge13 | Attention deficit hyperactivity disorder (ADHD) | 1=Disagree strongly  2=Disagree slightly  3=Neither disagree nor agree  4=Don’t know  5=Agree slightly  6=Agree strongly | Ordinal |
| 94 | Knowledge14 | Learning and intellectual disorders | 1=Disagree strongly  2=Disagree slightly  3=Neither disagree nor agree  4=Don’t know  5=Agree slightly  6=Agree strongly | Ordinal |
| 95 | Knowledge15 | Post-traumatic stress disorder (PTSD) | 1=Disagree strongly  2=Disagree slightly  3=Neither disagree nor agree  4=Don’t know  5=Agree slightly  6=Agree strongly | Ordinal |
| 96 | Knowledge16 | Self-harm and suicide | 1=Disagree strongly  2=Disagree slightly  3=Neither disagree nor agree  4=Don’t know  5=Agree slightly  6=Agree strongly | Ordinal |
| 97 | Knowledge17 | Epilepsy | 1=Disagree strongly  2=Disagree slightly  3=Neither disagree nor agree  4=Don’t know  5=Agree slightly  6=Agree strongly | Ordinal |
| Discipline | | | | |
| 98 | Discipline1 | How often did you shake or grab a child to get their attention? | 0=Never  1=Everyday  2=At least once a week  3=At least once a month  4=At least once a year | Ordinal |
| 99 | Discipline2 | How often did you spank, slap, smack, or swat a child? | 0=Never  1=Everyday  2=At least once a week  3=At least once a month  4=At least once a year | Ordinal |
| 100 | Discipline3 | How often did you use a paddle, hairbrush, belt, or other object to hit a child? | 0=Never  1=Everyday  2=At least once a week  3=At least once a month  4=At least once a year | Ordinal |
| 101 | Discipline4 | How often did you wash a child’s mouth out with soap, put hot sauce on their tongue, or something similar? | 0=Never  1=Everyday  2=At least once a week  3=At least once a month  4=At least once a year | Ordinal |
| 102 | Discipline5 | How often did you shout or yell at a child? | 0=Never  1=Everyday  2=At least once a week  3=At least once a month  4=At least once a year | Ordinal |
| 103 | Discipline6 | How often did you try to make a child feel ashamed or guilty? | 0=Never  1=Everyday  2=At least once a week  3=At least once a month  4=At least once a year | Ordinal |
| 104 | Discipline7 | When a child behaved badly, how often did you tell the child that they are lazy, sloppy, thoughtless, or some other name like that? | 0=Never  1=Everyday  2=At least once a week  3=At least once a month  4=At least once a year | Ordinal |
| 105 | Discipline8 | How often did you explain to a child what the rules are to try to prevent the child repeating misbehaviour? | 0=Never  1=Everyday  2=At least once a week  3=At least once a month  4=At least once a year | Ordinal |
| 106 | Discipline9 | How often did you put this child in time-out or send them to the head teacher? | 0=Never  1=Everyday  2=At least once a week  3=At least once a month  4=At least once a year | Ordinal |
| 107 | Discipline10 | How often did you give this child something else they might like to do instead of what they were doing wrong? | 0=Never  1=Everyday  2=At least once a week  3=At least once a month  4=At least once a year | Ordinal |
| 108 | Discipline11 | How often did you demonstrate the right thing to do for this child? | 0=Never  1=Everyday  2=At least once a week  3=At least once a month  4=At least once a year | Ordinal |
| Alcohol and substance use | | | | |
| 109 | Alcoholandsubs1 | How often do you have a drink containing alcohol? | 1=Never  1=Monthly or less  2=2 - 4 times per month  3=2 - 3 times per week  4=4+ times per week | Ordinal |
| 110 | Alcoholandsubs2 | How many units of alcohol do you drink on a typical day when you are drinking? Please estimate a number equivalent to bottles of beer, even if you are talking about drinking from the pot | 1=1-2  2=3-4  3=5-6  4=7-9  5=10+ | Ordinal |
| 111 | Alcoholandsubs3 | How often have you had 6 or more units if female, or 8 or more if male, on a single occasion in the last year? | 0=Never  1=Less than monthly  2=Monthly  3=Weekly  4=Daily or almost daily | Ordinal |
| 112 | Alcoholandsubs4 | How often during the last year have you found that you were not able to stop drinking once you had started? | 0=Never  1=Less than monthly  2=Monthly  3=Weekly  4=Daily or almost daily | Ordinal |
| 113 | Alcoholandsubs5 | How often during the last year have you failed to do what was normally expected from you because of your drinking? | 0=Never  1=Less than monthly  2=Monthly  3=Weekly  4=Daily or almost daily | Ordinal |
| 114 | Alcoholandsubs6 | How often during the last year have you needed an alcoholic drink in the morning to get yourself going after a heavy drinking session? | 0=Never  1=Less than monthly  2=Monthly  3=Weekly  4=Daily or almost daily | Ordinal |
| 115 | Alcoholandsubs7 | How often during the last year have you had a feeling of guilt or remorse after drinking? | 0=Never  1=Less than monthly  2=Monthly  3=Weekly  4=Daily or almost daily | Ordinal |
| 116 | Alcoholandsubs8 | How often during the last year have you been unable to remember what happened the night before because you had been drinking? | 0=Never  1=Less than monthly  2=Monthly  3=Weekly  4=Daily or almost daily | Ordinal |
| 117 | Alcoholandsubs9 | Have you or somebody else been injured as a result of your drinking? | 1=No  2=Yes, but not in the last year  3=Yes, during the last year | Nominal |
| 118 | Alcoholandsubs10 | Has a relative or friend, doctor or other health worker been concerned about your drinking or suggested that you cut down? | 1=No  2=Yes, but not in the last year  3=Yes, during the last year | Nominal |
| 119 | Alcoholandsubs11 | Have you ever used any of the following substances or drugs? | 0= None  1=Marijuana  2=Opioids/Strong pain killers  3=Tranquilizers/Valium/benzodiazepines  4=Fuel/glue/paint thinner  5=Cocaine /Amphetamine  6=Kath/Mira  7=Ecstasy/MDMA  8=Magic mushrooms/psilocybin  9=LSD | Nominal |

| **PREVIOUS VERSIONS OF THIS FORM:** | ***None.*** |
| --- | --- |
