# Supplementary material for: Factors associated with mental health stigma among teachers and caregivers of primary school children in Uganda
Source: BMC Public Health. 2025 Nov 4;25:3771. doi: 10.1186/s12889-025-25059-z (PMC12584251; doi:10.1186/s12889-025-25059-z)
Supplement: Supplementary file 2 — Supplementary Material 2. [file 12889_2025_25059_MOESM2_ESM.doc]

| **DOCUMENT:** | **DATA DICTIONARY** |
| --- | --- |
| **STUDY TITLE:** | **TREAT INTERACT: IMPLEMENTING A USER INVOLVED**  **EDUCATION- AND HEALTH SYSTEM INTERACTIVE TASK-**  **SHIFTING APPROACH FOR CHILD MENTAL HEALTH**  **PROMOTION IN UGANDA.** |
| **SURVEY GROUP** | **TEACHERS** |
| **INSTITUTIONS:** | **MAKERERE UNIVERSITY SCHOOL OF PUBLIC HEALTH, NORWEGIAN CENTER FOR VIOLENCE AND TRAUMATIC STRESS STUDIES, UNIVERSITY OF BERGEN, THE NORWEGIAN UNIVERSITY OF SCIENCE AND TECHNOLOGY AND THE NORWEGIAN INSTITUTE FOR OF PUBLIC HEALTH** |
| **BIOSTATISTICIAN** | **KALIBBALA DENNIS**  [**kalibbaladennis@gmail.com**](mailto:kalibbaladennis@gmail.com) **+256 (0) 777666152 /750631315** |
| **DATA MANAGER** | **OLET STEPHEN CHARLES**  [**stecho36@gmail.com**](mailto:stecho36@gmail.com) **+256 (0) 777824203, 0757433711** |
| **MAIN INVESTIGATORS:** | **1. JULIET NDIMWIBO BABIRYE**  **2. INGUNN ENGEBRETSEN**  **3. NORBERT SKOKAUSKAS**  **4. NORA BRAATHU**  **5. ESTHER KISAKYE**  **6. MUKISA MARJORIE KABATOORO**  **7. JOYCE SSERUNJOGI NALUGYA**  **8. HARRIET ABER**  **9. VILDE SKYLSTAD**  **10. MELF-JAKOB KÜHL**  **11. TORE WENTZEL-LARSEN**  **12. HARALD BÆKKELUND**  **13. ANE-MARTHE SOLHEIM SKAR** |
| **DATE** | **1^ST^ SEPTEMBER 2023** |

| **S/N** | **VARIABLES** | **DESCRIPTION** | **RESPONSE OPTIONS** | **TYPE OF VARIABLE** |
| --- | --- | --- | --- | --- |
|  | **Demographic information** | | |  |
| 1 | IDteacher | ID teacher | Numeric | Discrete |
| 2 | IDschool | ID school | Numeric | Discrete |
| 3 | Date | Note today’s date | Date | Ordinal |
| 4 | Age | How old are you now? | Numeric | Continuous |
| 5 | Sex | What is your sex? | 0=Male  1=Female | Nominal |
| 6 | Teachother | Do you teach in other schools, other than the current one you are at right now? | 0=No  1=Yes | Binary |
| 7 | Whichschools | Specify which schools | Text | String |
| 8 | Answered | Have you answered this questionnaire before for another school | 0=No  1=Yes | Binary |
|  | School Experience | | |  |
| 9 | Working | How many years have you been working as a teacher? | Numeric | Continuous |
| 10 | Currentschool | How many years have you been working at your current school? | Numeric | Continuous |
| 11 | Leveleduc | What is your highest completed level of education? | 1=O-level  2=A-level  3=Certificate  4=Diploma  5=Degree  6=Master degree  7=Post graduate diploma  8=Post graduate degree (Ph.D.) | Nominal |
| 12 | teachmost | What level do you teach most? | 0=Nursery Section  1=Primary 1  2=Primary 2  3=Primary 3  4=Primary 4  5=Primary 5  6=Primary 6  7=Primary 7 | Nominal |
| 13 | Subjectteach | Which subjects do you teach the most? | 1=Science  2= Mathematics  3= English  4= SST (social studies)  5=Other | Nominal |
| 14 | Notesubjectteach | Please specify | Text | String |
| 15 | Trainingcourse | Have you done a teacher’s training course? | 0=No  1=Yes | Binary |
| 16 | Subjecttrained | Which subjects were you trained to teach? | 1=Science  2= Mathematics  3= English  4= SST (social studies)  5=Other | Nominal |
| 17 | Notesubjecttrained | Please specify | Text | String |
|  | Mental health training | | |  |
| 18 | MHtraining | Have you received any mental health training before this session? | 0=No  1=During teacher's training  2=After teacher's training  3=Before teacher's training | Nominal |
| 19 | Orgprovided | If you have received any mental health training(s), please specify which organisation provided the training | 1=Ministry of education  2=Ministry of health  3= NGOs  4= Other | Nominal |
| 20 | Noteorgprovided | If you have received any mental health training(s), please specify | Text | String |
| 21 | MHTtopics | If you have received any mental health training(s), please specify what topics it covered in the training | Text | String |
| 22 | MHTduration | If you have received any mental health training(s), please specify how long was the training | Numeric | Continuous |
| 23 | MHTago | If you have received any mental health training(s), please specify how long ago was the training | 1=Less than a year ago  2=1 year ago  3=2 years ago  4=3 – 5 years ago  5=6 – 9 years ago  6=10 + years ago | Ordinal |
|  | Role | | |  |
| 24 | Position | What is your position in the school? | 1=Head teacher  2=Senior man teacher/Senior women teacher  3=Special Needs Teacher  4=Other | Nominal |
| 25 | Noteposition | Please Specify | Text | String |
| 26 | MHTrole | Are you currently involved in any mental health activities in your school? | 0=No  1=I am managing/overseeing mental health activities in the school  2=I am delivering the mental health interventions  3=I have no specific role in the mental health activities | Nominal |
|  | Referrals | | |  |
| 27 | Referredchild | Have you ever referred a child at school to the health system? | 0=No  1=Yes | Binary |
| 28 | MHproblem | If yes, have any of these referrals to the health system been because of a mental health problem? | 0=No  1=Yes | Binary |
| 29 | MHreason | If yes on Question 2: What problems have you referred children for? | 1=Depression  2=Stress  3=Schizophrenia  4=Bipolar Disorder  5=Attention deficit hyperactivity disorder (ADHD)  6=Learning and intellectual disorders  7=Post-traumatic stress disorder (PTSD)  8=Self-harm and suicide  9=Epilepsy  10=Alcohol related problem  11=Child used other substances  12=Other | Nominal |
| 30 | NoteMHreason | Please Specify | Text | String |
| 31 | Numberreferredmh | If yes on Question 2: How many children have you referred to the health system due to mental health concerns? | Numeric | Continuous |
| 32 | Numberreferredsu | If yes on Question 2: How many children have you referred to the health system due to alcohol or substance use? | Numeric | Continuous |
| 33 | Youngersu | If >0 on Question 5: How many of them were younger than 10 years old? | Numeric | Continuous |
|  | Discipline | | |  |
| 34 | Discipline1 | How often did you shake or grab a child to get their attention? | 1=Every day  2=At least once a week  3=At least once a month  4=At least once a year | Ordinal |
| 35 | Discipline2 | How often did you spank, slap, smack, or swat a child? | 1=Every day  2=At least once a week  3=At least once a month  4=At least once a year | Ordinal |
| 36 | Discipline3 | How often did you use a paddle, hairbrush, belt, or other object? | 1=Every day  2=At least once a week  3=At least once a month  4=At least once a year | Ordinal |
| 37 | Discipline4 | How often did you wash a child’s mouth out with soap, put hot sauce on their tongue, or something similar? | 1=Every day  2=At least once a week  3=At least once a month  4=At least once a year | Ordinal |
| 38 | Discipline5 | How often did you shout or yell at a child? | 1=Every day  2=At least once a week  3=At least once a month  4=At least once a year | Ordinal |
| 39 | Discipline6 | How often did you try to make a child feel ashamed or guilty? | 1=Every day  2=At least once a week  3=At least once a month  4=At least once a year | Ordinal |
| 40 | Discipline7 | When a child behaved badly, how often did you tell the child that they are lazy, sloppy, thoughtless, or some other name like that? | 1=Every day  2=At least once a week  3=At least once a month  4=At least once a year | Ordinal |
| 41 | Discipline8 | How often did you explain to a child what the rules are to try to prevent the child repeating misbehaviour? | 1=Every day  2=At least once a week  3=At least once a month  4=At least once a year | Ordinal |
| 42 | Discipline9 | How often did you put this child in time-out or send them to the head teacher? | 1=Every day  2=At least once a week  3=At least once a month  4=At least once a year | Ordinal |
| 43 | Discipline10 | How often did you give this child something else they might like to do instead of what they were doing wrong? | 1=Every day  2=At least once a week  3=At least once a month  4=At least once a year | Ordinal |
| 44 | Discipline11 | How often did you demonstrate the right thing to do for this child? | 1=Every day  2=At least once a week  3=At least once a month  4=At least once a year | Ordinal |
|  | Concerns about child mental health | | |  |
| 45 | Concerns1 | How many children at school have you ever had concerns about regarding their mental health? | Numeric | Continuous |
| 46 | Concerns2 | Did you refer these children to any of the following | 1=School nurse  2=Senior man or senior woman  3=Health care worker  4=Religious priest  5=Traditional healer  6=Others  7=I did not refer | Nominal |
| 47 | Noteconcerns2 | Please Specify | Text |  |
| 48 | Concerns3 | How many of those children did you have concerns about in the past three months? | 0=0  1=1  2=2  3=3  4=4  5=5 - 10  6=10+ | Ordinal |
| 49 | Concerns4 | How many of them did you give psychological support to | Numeric | Continuous |
| 50 | Concerns5 | How many of them did you refer for further care? | Numeric | Continuous |
| 51 | Concerns6 | What was the reason for referral? | 1=Depression  2=Stress  3=Schizophrenia  4=Bipolar Disorder  5=Attention deficit hyperactivity disorder (ADHD)  6=Learning and intellectual disorders  7=Post-traumatic stress disorder (PTSD)  8=Self-harm and suicide  9=Epilepsy  10=Other | Nominal |
| 52 | Noteconcerns6 | Please Specify | Text | String |
| 53 | Concerns7 | If no is ticked on question 5, what was the reason you did not refer any child, despite having been worried about their mental health? | 1=Parent did not show up  2=Child became better with counselling or talking therapy  3=Others | Nominal |
| 54 | NoteConcerns7 | Please Specify | Text | String |
|  | Personal Mental Health | | |  |
| 55 | Personal1 | Been able to concentrate on what you’re doing? | 1=Better than usual  2=Same as usual  3=Less than usual  4=Much less than usual | Ordinal |
| 56 | Personal2 | Lost much sleep over worry? | 1=Better than usual  2=Same as usual  3=Less than usual  4=Much less than usual | Ordinal |
| 57 | Personal3 | Felt that you are playing a useful part in home affairs? | 1=Better than usual  2=Same as usual  3=Less than usual  4=Much less than usual | Ordinal |
| 58 | Personal4 | Felt that you are playing a useful part at the school? | 1=Better than usual  2=Same as usual  3=Less than usual  4=Much less than usual | Ordinal |
| 59 | Personal5 | Felt capable of making decisions? | 1=Better than usual  2=Same as usual  3=Less than usual  4=Much less than usual | Ordinal |
| 60 | Personal6 | Felt constantly under strain? | 1=Better than usual  2=Same as usual  3=Less than usual  4=Much less than usual | Ordinal |
| 61 | Personal7 | Felt you couldn’t overcome your difficulties? | 1=Better than usual  2=Same as usual  3=Less than usual  4=Much less than usual | Ordinal |
| 62 | Personal8 | Been able to enjoy your normal day to day activities? | 1=Better than usual  2=Same as usual  3=Less than usual  4=Much less than usual | Ordinal |
| 63 | Personal9 | Been able to face up to your problems? | 1=Better than usual  2=Same as usual  3=Less than usual  4=Much less than usual | Ordinal |
| 64 | Personal10 | Been feeling unhappy or depressed? | 1=Better than usual  2=Same as usual  3=Less than usual  4=Much less than usual | Ordinal |
| 65 | Personal11 | Been losing confidence in yourself? | 1=Better than usual  2=Same as usual  3=Less than usual  4=Much less than usual | Ordinal |
| 66 | Personal12 | Been thinking of yourself as a worthless person? | 1=Better than usual  2=Same as usual  3=Less than usual  4=Much less than usual | Ordinal |
| 67 | Personal13 | Been feeling reasonably happy, all things considered? | 1=Better than usual  2=Same as usual  3=Less than usual  4=Much less than usual | Ordinal |
|  | Attitudes about Mental Health | | |  |
| 68 | Attitudes1 | A person who has received mental health treatment is just as intelligent as everyone else | 1=Strongly Disagree  2=Disagree  3=Disagree a little  4=Not sure  5=Agree a little  6=Agree  7=Strongly Agree | Ordinal |
| 69 | Attitudes2 | Someone who has received mental health treatment is just as trustworthy as everyone else | 1=Strongly Disagree  2=Disagree  3=Disagree a little  4=Not sure  5=Agree a little  6=Agree  7=Strongly Agree | Ordinal |
| 70 | Attitudes3 | It is acceptable that someone who has fully recovered from a mental illness can work as a teacher of young children in a public school. | 1=Strongly Disagree  2=Disagree  3=Disagree a little  4=Not sure  5=Agree a little  6=Agree  7=Strongly Agree | Ordinal |
| 71 | Attitudes4 | Receiving mental health treatment is a sign of personal failure or weakness. | 1=Strongly Disagree  2=Disagree  3=Disagree a little  4=Not sure  5=Agree a little  6=Agree  7=Strongly Agree | Ordinal |
| 72 | Attitudes5 | People with severe mental illness can have good quality of life, regardless of treatment. | 1=Strongly Disagree  2=Disagree  3=Disagree a little  4=Not sure  5=Agree a little  6=Agree  7=Strongly Agree | Ordinal |
| 73 | Attitudes6 | People with mental illness are to blame for their own condition. | 1=Strongly Disagree  2=Disagree  3=Disagree a little  4=Not sure  5=Agree a little  6=Agree  7=Strongly Agree | Ordinal |
| 74 | Attitudes7 | If my colleague told me he or she had a mental illness, I would still want to work with him or her. | 1=Strongly Disagree  2=Disagree  3=Disagree a little  4=Not sure  5=Agree a little  6=Agree  7=Strongly Agree | Ordinal |
| 75 | Attitudes8 | If I had neighbors with mental illness, I would move out of that neighborhood. | 1=Strongly Disagree  2=Disagree  3=Disagree a little  4=Not sure  5=Agree a little  6=Agree  7=Strongly Agree | Ordinal |
| 76 | Attitudes9 | If a person who had fully recovered from mental illness asked me for a letter of support to get employment, I would provide a reference. | 1=Strongly Disagree  2=Disagree  3=Disagree a little  4=Not sure  5=Agree a little  6=Agree  7=Strongly Agree | Ordinal |
| 77 | Attitudes10 | If I had a mental illness, I would not admit this to any of my friends for fear of being treated differently. | 1=Strongly Disagree  2=Disagree  3=Disagree a little  4=Not sure  5=Agree a little  6=Agree  7=Strongly Agree | Ordinal |
|  | Mental Health Knowledge | | |  |
| 78 | Mental1 | Most people with mental health problems want to work | 1=Disagree strongly  2=Disagree slightly  3=Neither disagree nor agree  4=Don’t know  5=Agree slightly  6=Agree strongly | Ordinal |
| 79 | Mental2 | If a friend had a mental health problem, I would advise him or her to get professional help | 1=Disagree strongly  2=Disagree slightly  3=Neither disagree nor agree  4=Don’t know  5=Agree slightly  6=Agree strongly | Ordinal |
| 80 | Mental3 | Medication can be an effective treatment for people with mental health problems | 1=Disagree strongly  2=Disagree slightly  3=Neither disagree nor agree  4=Don’t know  5=Agree slightly  6=Agree strongly | Ordinal |
| 81 | Mental4 | Psychological counselling can be an effective treatment for people with mental health problems | 1=Disagree strongly  2=Disagree slightly  3=Neither disagree nor agree  4=Don’t know  5=Agree slightly  6=Agree strongly | Ordinal |
| 82 | Mental5 | People with severe mental health problems can fully recover, with treatment | 1=Disagree strongly  2=Disagree slightly  3=Neither disagree nor agree  4=Don’t know  5=Agree slightly  6=Agree strongly | Ordinal |
| 83 | Mental6 | People with severe mental health problems can fully recover, without treatment | 1=Disagree strongly  2=Disagree slightly  3=Neither disagree nor agree  4=Don’t know  5=Agree slightly  6=Agree strongly | Ordinal |
| 84 | Mental7 | Most people with mental health problems go to a healthcare professional to get help | 1=Disagree strongly  2=Disagree slightly  3=Neither disagree nor agree  4=Don’t know  5=Agree slightly  6=Agree strongly | Ordinal |
|  | Type of mental illness | | |  |
| 85 | Mental8 | Depression | 1=Disagree strongly  2=Disagree slightly  3=Neither disagree nor agree  4=Don’t know  5=Agree slightly  6=Agree strongly  7=Haven’t heard of this condition | Ordinal |
| 86 | Mental9 | Stress | 1=Disagree strongly  2=Disagree slightly  3=Neither disagree nor agree  4=Don’t know  5=Agree slightly  6=Agree strongly  7=Haven’t heard of this condition | Ordinal |
| 87 | Mental10 | Schizophrenia | 1=Disagree strongly  2=Disagree slightly  3=Neither disagree nor agree  4=Don’t know  5=Agree slightly  6=Agree strongly  7=Haven’t heard of this condition | Ordinal |
| 88 | Mental11 | Bipolar disorder (manic depression) | 1=Disagree strongly  2=Disagree slightly  3=Neither disagree nor agree  4=Don’t know  5=Agree slightly  6=Agree strongly  7=Haven’t heard of this condition | Ordinal |
| 89 | Mental12 | Alcohol and other drug use disorders | 1=Disagree strongly  2=Disagree slightly  3=Neither disagree nor agree  4=Don’t know  5=Agree slightly  6=Agree strongly  7=Haven’t heard of this condition | Ordinal |
| 90 | Mental13 | Attention deficit hyperactivity disorder (ADHD) | 1=Disagree strongly  2=Disagree slightly  3=Neither disagree nor agree  4=Don’t know  5=Agree slightly  6=Agree strongly  7=Haven’t heard of this condition | Ordinal |
| 91 | Mental14 | Learning and intellectual disorders | 1=Disagree strongly  2=Disagree slightly  3=Neither disagree nor agree  4=Don’t know  5=Agree slightly  6=Agree strongly  7=Haven’t heard of this condition | Ordinal |
| 92 | Mental15 | Post-traumatic stress disorder (PTSD) | 1=Disagree strongly  2=Disagree slightly  3=Neither disagree nor agree  4=Don’t know  5=Agree slightly  6=Agree strongly  7=Haven’t heard of this condition | Ordinal |
| 93 | Mental16 | Self harm and suicide | 1=Disagree strongly  2=Disagree slightly  3=Neither disagree nor agree  4=Don’t know  5=Agree slightly  6=Agree strongly  7=Haven’t heard of this condition | Ordinal |
| 94 | Mental17 | Epilepsy | 1=Disagree strongly  2=Disagree slightly  3=Neither disagree nor agree  4=Don’t know  5=Agree slightly  6=Agree strongly  7=Haven’t heard of this condition | Ordinal |
|  | Questions about gender norms | | |  |
| 95 | Gender1 | It is important that sons have more education than daughters | 0=Disagree  1=Agree | Binary |
| 96 | Gender2 | Daughters should not be sent to school if they are needed to help at home | 0=Disagree  1=Agree | Binary |
| 97 | Gender3 | The most important reason that sons should be more educated than daughters is so that the sons can better look after their parents when they are older | 0=Disagree  1=Agree | Binary |
| 98 | Gender4 | If there is limited amount of money to pay for school fees, it should be spent on the sons first | 0=Disagree  1=Agree | Binary |
| 100 | Gender5 | It is a woman’s role to take good care of the children and not worry about other people’s affairs | 0=Disagree  1=Agree | Binary |
| 101 | Gender6 | Women should leave politics to men | 0=Disagree  1=Agree | Binary |
| 102 | Gender7 | A woman has to have a husband or sons or some other male relative to protect her | 0=Disagree  1=Agree | Binary |
| 103 | Gender8 | The only thing a woman can really rely on in her old age is her sons | 0=Disagree  1=Agree | Binary |
| 104 | Gender9 | A good woman never questions her husband’s opinions out loud, even if she is not sure she agrees with them | 0=Disagree  1=Agree | Binary |
| 105 | Gender10 | When it is a question of children’s health, it is best to do whatever the father wants | 0=Disagree  1=Agree | Binary |
| 106 | Gender11 | I would like my daughter to be able to work outside the home so she can support herself if necessary | 0=Disagree  1=Agree | Binary |
|  | Implementation Quality Questionnaire | | |  |
|  | Theme One | | |  |
| 107 | Implementation1 | You are positive about the introduction of the mental health training programme at your school | 1=Strongly Disagree  2=Disagree  3=Disagree a little  4=Neither disagree or agree  5=Agree a little  6=Agree  7=Strongly Agree | Ordinal |
| 108 | Implementation2 | Other people at the school will be opposed to the introduction of the mental health training programme | 1=Strongly Disagree  2=Disagree  3=Disagree a little  4=Neither disagree or agree  5=Agree a little  6=Agree  7=Strongly Agree | Ordinal |
| 109 | Implementation3 | Mental health promotion activities are needed in Ugandan schools today | 1=Strongly Disagree  2=Disagree  3=Disagree a little  4=Neither disagree or agree  5=Agree a little  6=Agree  7=Strongly Agree | Ordinal |
| 110 | Implementation4 | You have sufficient expertise (e.g. skills/knowledge) to successfully implement the mental health training programme | 1=Strongly Disagree  2=Disagree  3=Disagree a little  4=Neither disagree or agree  5=Agree a little  6=Agree  7=Strongly Agree | Ordinal |
| 111 | Implementation5 | It will be possible to carry out the mental health training programme as planned | 1=Strongly Disagree  2=Disagree  3=Disagree a little  4=Neither disagree or agree  5=Agree a little  6=Agree  7=Strongly Agree | Ordinal |
| 112 | Implementation6 | The mental health training programme will require too much of the teachers(e.g. time or effort) | 1=Strongly Disagree  2=Disagree  3=Disagree a little  4=Neither disagree or agree  5=Agree a little  6=Agree  7=Strongly Agree | Ordinal |
| 113 | Implementation7 | The training and supervisions of teachers in mental health training programme will occur often enough | 1=Strongly Disagree  2=Disagree  3=Disagree a little  4=Neither disagree or agree  5=Agree a little  6=Agree  7=Strongly Agree | Ordinal |
| 114 | Implementation8 | The contents of the mental health training programme seem convenient and easily applicable | 1=Strongly Disagree  2=Disagree  3=Disagree a little  4=Neither disagree or agree  5=Agree a little  6=Agree  7=Strongly Agree | Ordinal |
|  | Theme Two | | |  |
| 115 | Implementation9 | The mental health training programme will meet the challenges of this school | 1=Strongly Disagree  2=Disagree  3=Disagree a little  4=Neither disagree or agree  5=Agree a little  6=Agree  7=Strongly Agree | Ordinal |
| 116 | Implementation10 | Themental health training programme will fit with existing expectations, standards, and needs in the school | 1=Strongly Disagree  2=Disagree  3=Disagree a little  4=Neither disagree or agree  5=Agree a little  6=Agree  7=Strongly Agree | Ordinal |
| 117 | Implementation11 | The mental health training programme will strengthen teachers' ability to promote pupils' mental health | 1=Strongly Disagree  2=Disagree  3=Disagree a little  4=Neither disagree or agree  5=Agree a little  6=Agree  7=Strongly Agree | Ordinal |
| 118 | Implementation12 | The mental health training programme is done for Western, rich countries and does not fit in a Ugandan school context | 1=Strongly Disagree  2=Disagree  3=Disagree a little  4=Neither disagree or agree  5=Agree a little  6=Agree  7=Strongly Agree | Ordinal |
| 119 | Implementation13 | The currentrelationship between staff at the school and management (head teacher) should be improved. | 1=Strongly Disagree  2=Disagree  3=Disagree a little  4=Neither disagree or agree  5=Agree a little  6=Agree  7=Strongly Agree | Ordinal |
| 120 | Implementation14 | There is good communication within the school | 1=Strongly Disagree  2=Disagree  3=Disagree a little  4=Neither disagree or agree  5=Agree a little  6=Agree  7=Strongly Agree | Ordinal |
| 121 | Implementation15 | There is agreement on teaching standards and values in the school | 1=Strongly Disagree  2=Disagree  3=Disagree a little  4=Neither disagree or agree  5=Agree a little  6=Agree  7=Strongly Agree | Ordinal |
|  | Theme Three | | |  |
| 122 | Implementation16 | It will be easier to cooperate with other district services (e.g. health centers) after the introduction of the mental health training programme? | 1=Strongly Disagree  2=Disagree  3=Disagree a little  4=Neither disagree or agree  5=Agree a little  6=Agree  7=Strongly Agree | Ordinal |
| 123 | Implementation17 | There has been given enough information about the mental health training programme | 1=Strongly Disagree  2=Disagree  3=Disagree a little  4=Neither disagree or agree  5=Agree a little  6=Agree  7=Strongly Agree | Ordinal |
| 124 | Implementation18 | Those who conveyed that themental health training programme should be carried out were enthusiastic and had faith in it | 1=Strongly Disagree  2=Disagree  3=Disagree a little  4=Neither disagree or agree  5=Agree a little  6=Agree  7=Strongly Agree | Ordinal |
| 125 | Implementation19 | There will beclear indications that the school is prioritizing the mental health training programme | 1=Strongly Disagree  2=Disagree  3=Disagree a little  4=Neither disagree or agree  5=Agree a little  6=Agree  7=Strongly Agree | Ordinal |
| 126 | Implementation20 | Not enough time will be set aside to work on the mental health training programme | 1=Strongly Disagree  2=Disagree  3=Disagree a little  4=Neither disagree or agree  5=Agree a little  6=Agree  7=Strongly Agree | Ordinal |
| 127 | Implementation21 | The introduction of the mental health training programme will beat the expense of other important tasks | 1=Strongly Disagree  2=Disagree  3=Disagree a little  4=Neither disagree or agree  5=Agree a little  6=Agree  7=Strongly Agree | Ordinal |
| 128 | Implementation22 | The mental health training programme will be implemented at a good timefor your school | 1=Strongly Disagree  2=Disagree  3=Disagree a little  4=Neither disagree or agree  5=Agree a little  6=Agree  7=Strongly Agree | Ordinal |
| 129 | Implementation23 | The school will be ready to introduce the mental health training programme | 1=Strongly Disagree  2=Disagree  3=Disagree a little  4=Neither disagree or agree  5=Agree a little  6=Agree  7=Strongly Agree | Ordinal |
| 130 | Implementation24 | Staff at this school will be involved in the planning process | 1=Strongly Disagree  2=Disagree  3=Disagree a little  4=Neither disagree or agree  5=Agree a little  6=Agree  7=Strongly Agree | Ordinal |
| 131 | Implementation25 | School management will become involved in the planning process | 1=Strongly Disagree  2=Disagree  3=Disagree a little  4=Neither disagree or agree  5=Agree a little  6=Agree  7=Strongly Agree | Ordinal |
| 132 | Implementation26 | You will be given the chance to provide feedback on the mental health training programme along the way | 1=Strongly Disagree  2=Disagree  3=Disagree a little  4=Neither disagree or agree  5=Agree a little  6=Agree  7=Strongly Agree | Ordinal |

| **PREVIOUS VERSIONS OF THIS FORM:** | ***None.*** |
| --- | --- |
