# Supplementary figures and images for: Factors associated with mental health stigma among teachers and caregivers of primary school children in Uganda
Source: BMC Public Health. 2025 Nov 4;25:3771. doi: 10.1186/s12889-025-25059-z (PMC12584251; doi:10.1186/s12889-025-25059-z)

Mental Health Stigma

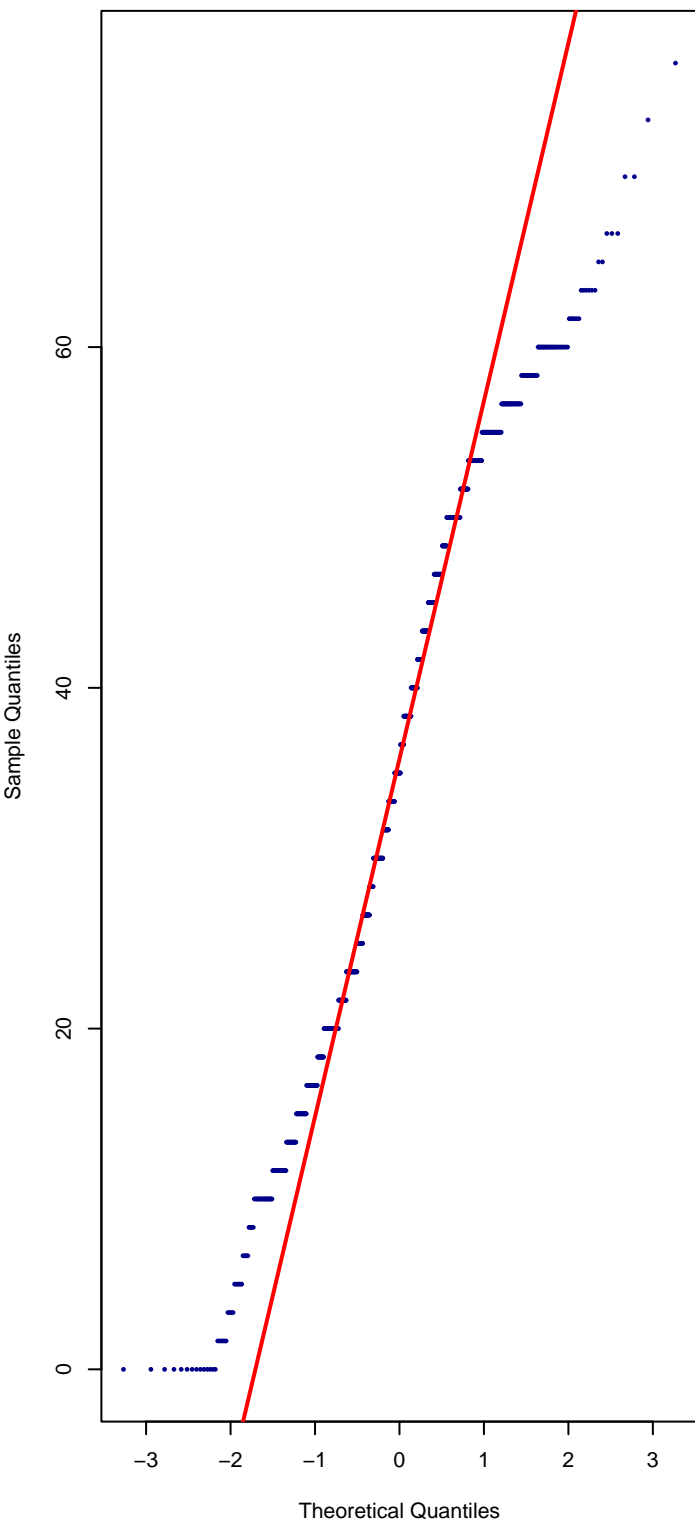

Mental Health Knowledge

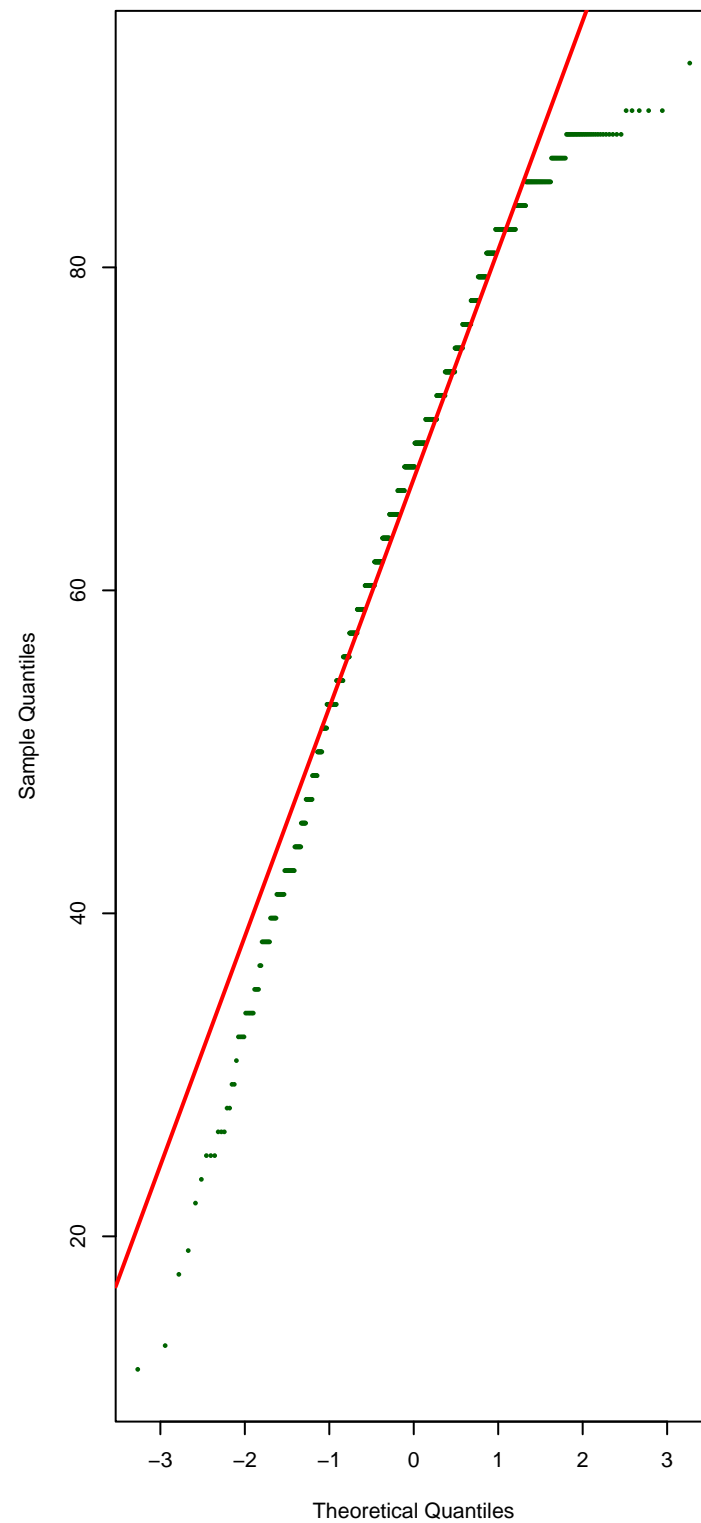

Psychological Aggression

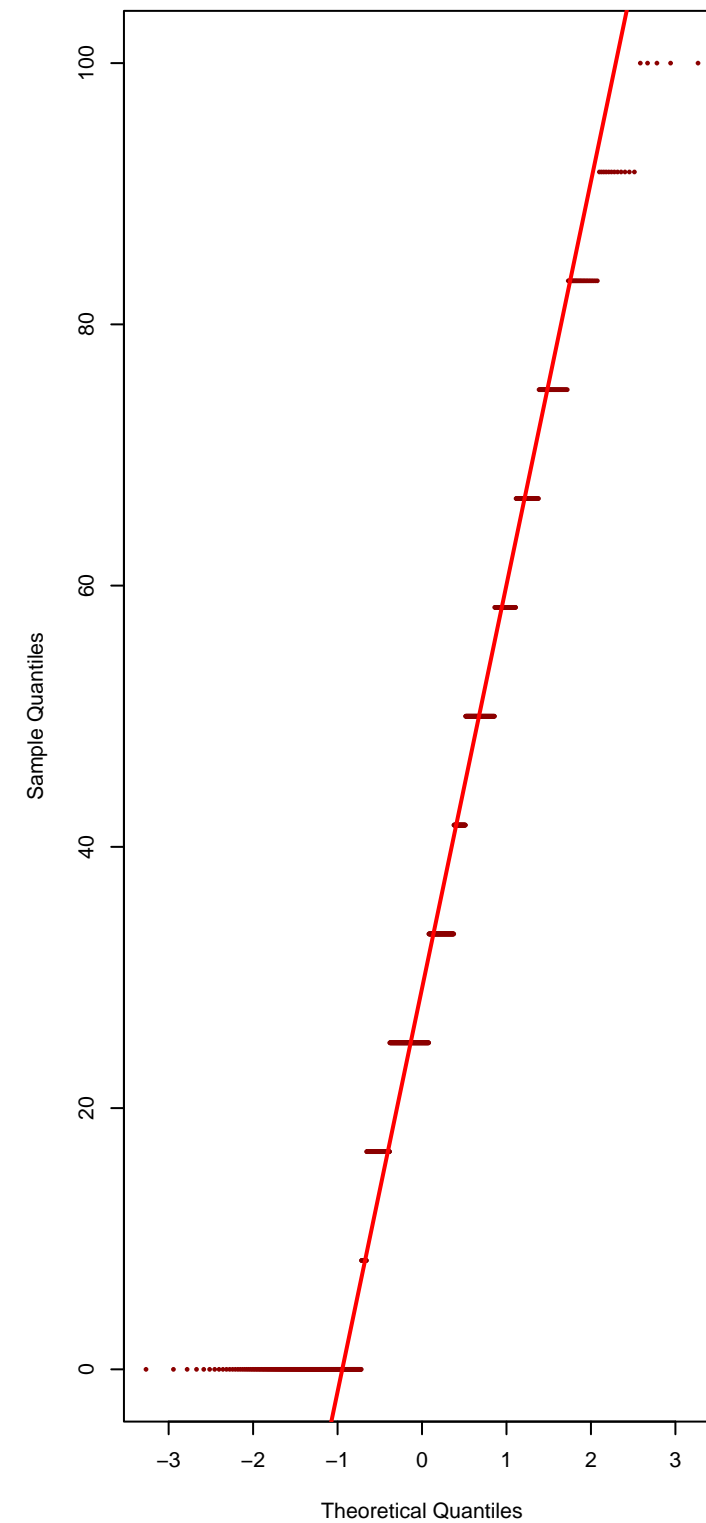

Supplement: Supplementary file 3 — Supplementary Material 3. [file 12889_2025_25059_MOESM3_ESM.pdf]
